# Effect of Outpatient Rehabilitation on Functional Mobility After Single Total Knee Arthroplasty: A Randomized Clinical Trial

**DOI:** 10.1001/jamanetworkopen.2020.16571

**Published:** 2020-09-17

**Authors:** Chinghui Jean Hsieh, Gerben DeJong, Michele Vita, Alexander Zeymo, Sameer Desale

**Affiliations:** 1 Agency for Healthcare Research and Quality, Department of Health and Human Services, Rockville, Maryland; 2 MedStar National Rehabilitation Hospital, Washington, District of Columbia; 3 Department of Rehabilitation Medicine, Georgetown University School of Medicine, Washington, District of Columbia; 4 MedStar National Rehabilitation Network, Washington, District of Columbia; 5 MedStar Health Research Institute, Hyattsville, Maryland

## Abstract

**Importance:**

Even without evidence, rehabilitation practitioners continue to introduce new interventions to enhance the mobility outcomes for the increasing population with a recent total knee arthroplasty (TKA).

**Objective:**

To compare post-TKA functional mobility outcomes among 3 newly developed physical therapy protocols with a standard-of-care post-TKA rehabilitation protocol.

**Design, Setting, and Participants:**

This randomized clinical trial included 4 study arms implemented in 15 outpatient clinics within a single health system in the Baltimore, Maryland, and Washington, District of Columbia, region from October 2013 to April 2017. Participants included patients who underwent elective unilateral TKA, were aged 40 years and older, and began outpatient physical therapy within 24 days after TKA. A total of 505 patients were screened and 386 participants were enrolled. Patients provided informed consent and were randomly assigned to 1 of 4 groups. Blinding patients and treating therapists was not feasible owing to the nature of the intervention. Analysis was conducted under the modified intent-to-treat principle from October 2017 to May 2019.

**Interventions:**

The control group used a standard recumbent bike for 15 to 20 minutes each session. Interventions used 1 of 3 modalities for 15 to 20 minutes each session: (1) a body weight-adjustable treadmill, (2) a patterned electrical neuromuscular stimulation device, or (3) a combination of the treadmill and electrical neuromuscular stimulation.

**Main Outcomes and Measures:**

Outcomes included the Activity Measure for Post-acute Care basic mobility score, a patient-reported outcome measure, and the 6-minute walk test. Outcomes were measured at baseline, monthly, and on discharge from outpatient therapy.

**Results:**

Data from 363 patients (mean [SD] age, 63.4 [7.9] years; 222 [61.2%] women) were included in the final analysis, including 92 participants randomized to the control group, 91 participants randomized to the treadmill group, 90 participants randomized to the neuromuscular stimulation device group, and 90 participants randomized to the combination intervention group. Activity Measure for Post-acute Care scores at discharge were similar across groups, ranging from 61.1 to 61.3 (*P* = .99) with at least 9.0 points improvement (*P* = .80) since baseline. The distances as measured by the 6-minute walking test were not statistically different across groups (range, 382.9-404.5 m; *P* = .60).

**Conclusions and Relevance:**

This randomized clinical trial found no statistically or clinically significant differences in outcomes across the 4 arms. Because outcomes were similar among arms, clinicians should instead consider relative cost in tailoring TKA rehabilitation.

**Trial Registration:**

ClinicalTrials.gov Identifier: NCT02426190

## Key Points

**Question:**

Are there short-term clinically significant rehabilitation outcome differences following total knee arthroplasty among patients in a standard rehabilitation care group and patients in any of 3 intervention groups using different types of equipment?

**Findings:**

This randomized clinical trial compared post–total knee arthroplasty functional mobility on discharge from outpatient rehabilitation among 363 patients who were randomly assigned to 1 of 4 groups using different types of equipment (ie, a recumbent bike, a body weight–adjustable treadmill, a patterned electrical neuromuscular stimulation device, and a combination of a body weight–adjustable treadmill and a patterned electrical neuromuscular stimulation device). The Activity Measure for Post-Acute Care scores (measuring functional mobility) and ambulatory distances (measuring functional capacity) at discharge were not statistically different across groups.

**Meaning:**

This randomized clinical trial found no statistically significant differences in functional outcomes on discharge based on the type of equipment used during patients’ post–total knee arthroplasty outpatient rehabilitation.

## Teaser

This randomized clinical trial compares outpatient rehabilitation for total knee arthroplasty among patients receiving 4 kinds of physical therapy.

## Introduction

The number of total knee arthroplasty (TKA) procedures has increased significantly in recent decades owing to both increased demand and supply.^1^ On the demand side is an increasing population with increasing longevity and rates of obesity contributing to the increased prevalence of knee osteoarthritis. On the supply side is an increase in the number of orthopedic surgeons performing TKAs, increasingly using less-invasive procedures.^2^

Payment policy changes in the US over the past decade have shifted post-TKA rehabilitation mainly to home health and outpatient settings. Patients often seek faster functional recovery in terms of community mobility (ie, the ability to ambulate ≥200 m, safely navigate curbs, use alternating gait up and down 2 sets of stairs using 1 handrail, and transfer in and out of cars or chairs) so that they can return to their normal routines quickly, particularly among working-aged individuals.^3,4,5,6,7,8,9,10,11,12^

Numerous studies have shown that rehabilitation is associated with improved post-TKA outcomes, whether comparing rehabilitation received at different settings or examining different physical therapy (PT) protocols.^13,14,15,16,17,18,19,20,21,22,23^ This typically requires active patient engagement by incorporating weight bearing exercise, active range of motion, and gait training. However, patients’ fear of falling and postsurgical pain accompanying weight bearing and range of motion exercises often limit patient’s ability to fully participate in PT and thus prolong a patient’s recovery.

Previous studies^24,25,26,27,28^ have shown that supporting a portion of a patient’s body weight during therapy can help mitigate pain and facilitate a patient’s involvement in more aggressive therapy. Similarly, studies using harnessed body weight–support systems have shown positive associations with functional gain.^24,25,26,27,28^ This randomized clinical trial used a Food and Drug Administration–cleared treadmill using patented National Aeronautics and Space Administration technology to unload a proportion of body weight during therapy without any harness or straps. The treadmill provides precise partial weight bearing that can be adjusted as patients progress over time. Studies using this equipment have shown positive impact on rehabilitation outcomes among children with cerebral palsy and patients undergoing knee surgery and Achilles tendon repair.^29,30,31^

Research has also demonstrated the complementary effectiveness of incorporating electrical stimulation in PT for postoperative neuromuscular re-education.^32,33,34,35,36,37^ Neuromuscular stimulation has historically been used to help manage pain, relieve muscle spasms, increase range of motion, prevent muscle disuse atrophy, increase circulation, and re-educate muscles. This study examined using an electrical neuromuscular stimulation device that uses a patterned wave form mimicking the firing pattern of muscles during a given activity to better facilitate neuromuscular re-education during therapy.

The study compared post-TKA rehabilitation outcomes among 3 newly developed PT protocols and a traditional protocol that uses a recumbent bike serving as a control arm among patients with a unilateral TKA in outpatient settings. The 3 new interventions included (1) a body weight–adjustable treadmill, (2) a patterned electrical neuromuscular stimulation (PENS) device, and (3) both together.

## Methods

### Trial Design

This study was a randomized, 4-arm parallel-group clinical trial comparing the rehabilitation outcomes among 3 intervention groups and a control group among patients who underwent TKA. This study was conducted in 15 outpatient clinics within a single rehabilitation network across the Baltimore, Maryland, and Washington, District of Columbia, region (Trial Protocol in Supplement 1). The study received institutional review board approval by the MedStar Health Research Institute institutional review board. A written informed consent was obtained from each participant. This study is reported following the Consolidated Standards of Reporting Trials (CONSORT) reporting guideline.

### Participants

Enrollment started in October 2013; the study ended in April 2017 when the last patients concluded their PT course. Eligible individuals for the study were those who (1) underwent an elective unilateral TKA and initiated outpatient PT within 24 days after TKA; (2) were aged 40 years or older; and (3) weighed less than 300 lb (to convert to kilograms, multiply by 0.45), owing to the body weight–adjustable treadmill weight limitation.

Participants were excluded if they (1) underwent any lower extremity joint replacement procedure less than 1 year prior to the current TKA; (2) were in litigation related to injury or disease associated with their current TKA; (3) had a recent medical history of neurologic disorders, rheumatoid arthritis, or gout; (4) were under active cancer treatment with history of malignant neoplasm in lower extremities or had recent evidence of signs or symptoms of cancer, chemotherapy, or radiation; (5) were unable to proceed or continue the planned outpatient program because of complications, such as wound infection, related to the TKA procedure or required manipulation under anesthesia due to knee stiffness after TKA^38^; and (6) had received more than 2 weeks of other postacute services prior to outpatient PT.

### Interventions

Each rehabilitation treatment session over the course of 8 to 12 weeks (2-3 times per week) consisted of 3 phases: an exercise and conditioning phase (15-20 minutes), a hands-on therapy and treatment phase (30-40 minutes), and a final pain management and edema control phase (15 minutes). The study’s control intervention and the 3 new interventions were used in the exercise and conditioning phase.

The exercise and conditioning phase seeks to increase blood flow and pliability of the tissue surrounding the surgical joint to the following hands-on therapy phase. Patients in the control group used a standard recumbent bike.

Intervention group 1 used a body weight–adjustable treadmill during the exercise phase to unload partial body weight when walking on the treadmill. Physical therapists identified the threshold body weight unloading that minimized pain and allowed patients to move freely while on the treadmill. Over time, physical therapists decreased body weight support as tolerated. Physical therapists also determined the appropriate walking speed allowing patients to maintain a proper gait pattern while on the treadmill.

Intervention group 2 used PENS on the leg that underwent TKA while using a recumbent bike during the exercise phase. A PENS unit supports early restoration of agonist/antagonist muscular timing patterns to encourage neuromuscular re-education following a TKA.

Intervention group 3 used both the body weight–adjustable treadmill and PENS during the initial phase. The combination simultaneously unloaded a proportion of patient’s body weight and facilitated the proper muscle recruitment pattern during ambulation.

The hands-on and treatment phase addressed strengthening, neuromuscular re-education, and manual therapy. Designed by physical therapists, this phase was tailored to individual patient needs and functional goals. It was typically a 1-on-1 format working directly with a physical therapist.

The final phase provided transition from exertion to rest after an intensive therapy session. Physical therapists sought to minimize secondary injury and loss of progress through pain and inflammation management prior to finishing the treatment session.

All physical therapists underwent a rigorous 1-day in-person training session followed by the study principal investigator’s (C.J.H.) visits to each clinic and regular conference calls (biweekly to monthly) to assure that the study’s standardized protocol was followed.

### Outcomes

The study’s primary outcome measures were the Activity Measure for Post-acute Care (AM-PAC)^39,40,41,42^ basic mobility score and the 6-minute walk test. Both were measured at initial evaluation, monthly, and at discharge from outpatient PT. The AM-PAC is a patient-reported instrument to measure functional levels in 3 domains: basic mobility, daily activity, and applied cognition. For purpose of the study, only the basic mobility domain was measured. This study used the AM-PAC paper short form designed for outpatient settings. The short form consists of 18 questions and produces a raw score (range, 18-72) transformed into a score ranging from 29.41 to 80.30 based on item degree of difficulty. Higher transformed scores denote higher functional mobility (ie, limited indoor mobility, ≤51.9; enhanced indoor mobility, 52-65.9; and outdoor mobility, 66-84).^40^

The 6-minute walk test is a measure of functional capacity developed to evaluate walking endurance among patients aged 60 to 90 years.^43,44,45,46,47,48^ The test has been used as a performance-based measure in various populations, including healthy older adults, patients recovering from stroke, and patients undergoing knee or hip arthroplasty.^47,49,50,51,52,53,54,55,56,57,58^ The 6-minute walk test measures the distance an individual can walk in 6 minutes on a hard, flat surface, with any assisting device allowed. Other information related to TKA or post-TKA rehabilitation was also captured, including use of an assistive device, pain medications, and weight-bearing status.

### Sample Size

The study power was estimated using the Power Analysis and Sample Size software, 2008 version (NCSS Statistical Software). The primary outcome was the basic mobility domain of the AM-PAC. The power calculations were based on primary hypotheses using *t* test. Power was set at 80%, while 2-sided α level was set at .016, using Bonferroni correction. Based on the results of previous studies and recommendations from the AM-PAC development group, we used a mean difference of 4 points with an SD of 8 in the domains of basic mobility as a clinically meaningful change from baseline to discharge to calculate sample size for the study.^39,40,41,42^ Minimum sample size required for each group was 90 patients.

### Randomization

Randomization occurred immediately after a participant provided informed consent at each study clinic. Participants were randomly assigned to the control group or 1 of the intervention groups using a randomized permuted block design of block size 8.^59^ Each participant’s group assignment was based on a site-specific sequentially numbered study ID on enrollment. Randomization was stratified based on study site to address potential center effect or bias. This helped to mitigate biases that might stem from differences in patient populations, care management, and other contextual factors that may be unique to an individual study site.

### Blinding

Study participants and treating physical therapists were not blinded to participant’s group assignment. To mitigate potential bias caused by non-blindness, we chose AM-PAC and 6-minute walk test as our 2 objective primary outcome measures for their excellent interrater reliability, external validity, and minimal vulnerability to rater bias.^41,42,47,60,61,62,63^

### Statistical Analysis

The study examined descriptive statistics, frequency distributions, and graphic plots of variables to detect any data errors, outliers, number and pattern of missing data, and normality of distributions. Baseline study population characteristics were presented as means (with SDs) or proportions by treatment group. The difference in means among 4 groups was compared using analysis of variance; the difference in percentage was compared using the χ^2^ test. Analysis was conducted using modified intent-to-treat. Missing outcome data were addressed using multiple imputation and the last-observation-carried forward.^64^ The means (SD) of AM-PAC and 6-minute walking test were calculated at baseline, monthly, and discharge. In addition, linear mixed models were used to estimate treatment effects between outcomes, and treatment groups controlling for covariates, such as sex, age, body mass index, employment, and course of outpatient therapy, to address potential patient-level random effects. Models were estimated using an initial-to-discharge difference-in-difference analysis and in a repeated measure approach with intermediate evaluations at 1 and 2 months after initial evaluation. All analysis was performed in R statistical software version 3.4 (R Project for Statistical Computing) using the *lme4*, *R2wd*, *doBy*, and *ggplot2* packages. Data were analyzed from October 2017 to May 2019.

## Results

### Recruitment

A total of 505 patients who underwent TKA were screened for eligibility, among whom 45 did not meet the inclusion criteria and 74 declined to participate. The remaining 386 eligible patients agreed to participate. Participant flow is presented in the Figure. Among these, 95 patients were randomized to the control group, 96 patients were randomized to intervention group 1, 96 patients were randomized to intervention group 2, and 99 patients were randomized to intervention group 3. Data from 23 patients were excluded from the analysis owing to insufficient data and withdrawal from the study. Consequently, data from 363 patients, including 92 patients in the control group, 91 patients in intervention group 1, 90 patients in intervention group 2, and 90 patients in intervention group 3, were included in the final analysis.

**Figure.**
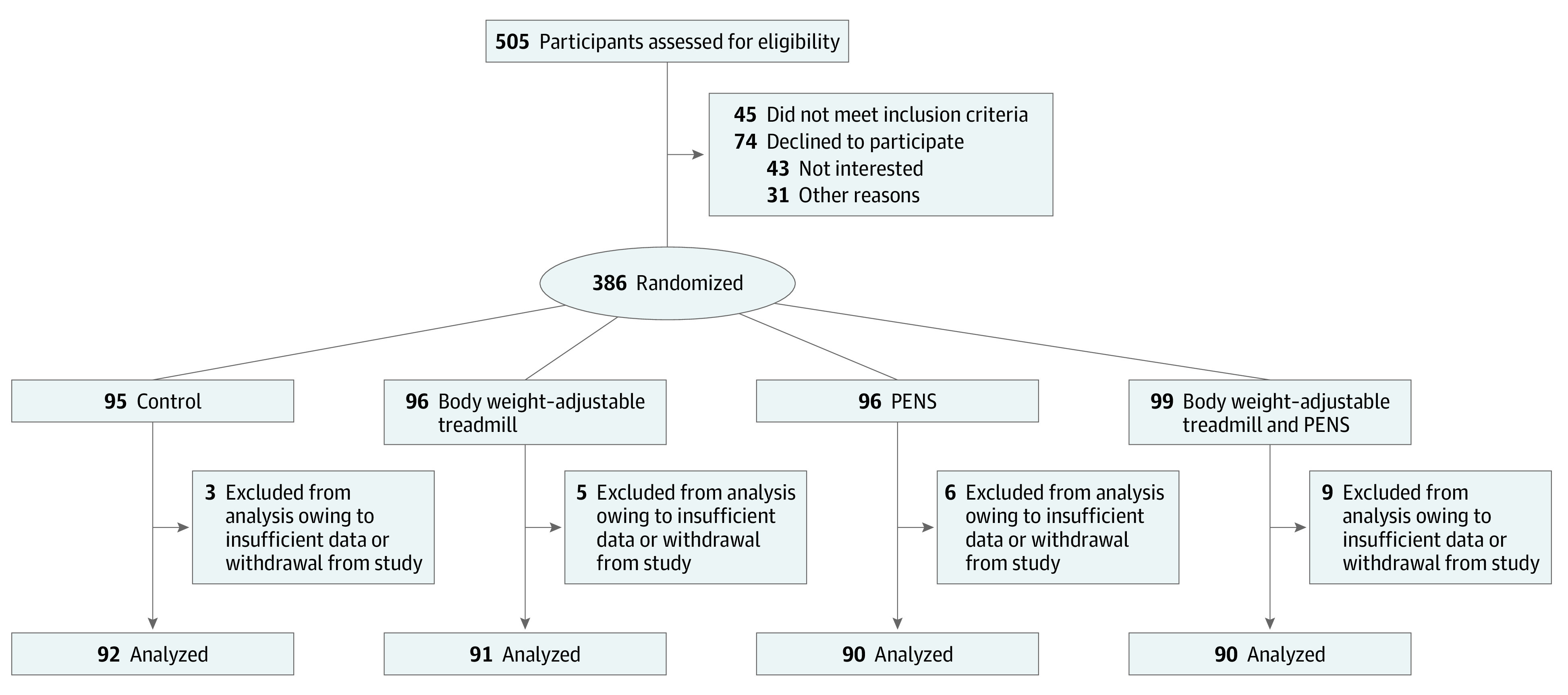
Participant Recruitment Flow Other reasons to decline to participate included insurance coverage issues, financial concerns (eg, no insurance, high co-payment), time commitment, requiring particular treatment protocols causing compliance concerns, and relocation. Reasons for exclusion from analysis included incomplete or insufficient data and withdrawal from study. PENS indicates patterned electrical neuromuscular stimulation.

### Baseline Data

Overall, patients did not differ across groups (Table1 and Table 2): the mean (SD) age was 63.4 (7.9) years old, 222 (61.2%) were women, 244 (67.2%) were White, 183 (50.4%) had at least a bachelor’s degree, and 361 (99.4%) were living at home. Most patients were overweight (108 patients [29.8%]) or obese (200 patients [55.1%]). Most had private insurance (220 patients [60.6%]) or Medicare (117 patients [32.3%]). Most patients had their TKA and rehabilitation performed within the study’s host health system (267 patients [73.6%]). The control group had a higher percentage of patients who had their left knee replaced (60 patients [65.2%]) compared with the other groups (intervention group 1:40 patients [44.0%]; intervention group 2: 41 patients [45.6%]; intervention group 3: 44 patients [48.9%]). Approximately one-fifth of patients had a previous TKA (63 patients [17.4%]) or a hip replacement (14 patients [3.9%]). The median (interquartile range) TKA length of stay in acute care was 3 (2-3) days.

**Table 1.** Study Group Characteristics

Characteristic	No. (%)			
	Control (n = 92)^a^	Intervention		
		Group 1 (n = 91)^b^	Group 2 (n = 90)^c^	Group 3 (n = 90)^b^^,^^c^
Age, mean (SD), y	62.8 (8.3)	64.9 (7.7)	62.9 (8.0)	62.7 (7.7)
Women	53 (57.6)	58 (63.7)	58 (64.4)	53 (58.9)
Race				
Black	25 (27.2)	23 (25.3)	26 (28.9)	29 (32.2)
White	63 (68.5)	63 (69.2)	62 (68.9)	56 (62.2)
Other	4 (4.3)	5 (5.5)	2 (2.2)	5 (5.6)
Education				
<High school	3 (3.3)	6 (6.6)	4 (4.4)	3 (3.3)
High school or GED	14 (15.2)	15 (16.5)	17 (18.9)	25 (27.8)
Some college	25 (27.2)	22 (24.2)	25 (27.8)	21 (23.3)
Bachelor’s degree	28 (30.4)	28 (30.8)	24 (26.7)	22 (24.4)
≥Graduate degree	22 (23.9)	20 (22.0)	20 (22.2)	19 (21.1)
Employment status				
Full-time	48 (52.2)	31 (34.1)	47 (52.2)	44 (48.9)
Part-time	5 (5.4)	2 (2.2)	5 (5.6)	7 (7.8)
None	6 (6.5)	15 (16.5)	9 (10.0)	7 (7.8)
Retired	33 (35.9)	43 (47.3)	29 (32.2)	32 (35.6)
Primary insurance payer				
Private or commercial	60 (65.2)	49 (53.8)	59 (65.6)	52 (57.8)
Medicare	28 (30.4)	31 (34.1)	26 (28.9)	32 (35.6)
Medicaid	2 (2.2)	9 (9.9)	4 (4.4)	5 (5.6)
Other	1 (1.1)	2 (2.2)	1 (1.1)	0
None	1 (1.1)	0	0	1 (1.1)
Living at home at initial evaluation	92 (100)	90 (98.9)	90 (100)	89 (98.9)
BMI				
Mean (SD)	31.5 (5.8)	31.2 (6.4)	32.2 (6.5)	31.41 (5.7)
Underweight	1 (1.1)	0	1 (1.1)	0
Within reference range	9 (9.8)	17 (19.1)	12 (13.6)	11 (12.2)
Overweight	31 (33.7)	24 (27.0)	19 (21.6)	34 (37.8)
Obese	51 (55.4)	48 (53.9)	56 (63.6)	45 (50.0)

Abbreviations: BMI, body mass index (calculated as weight in kilograms divided by height in meters squared); GED, general education diploma.

^a^ Used a recumbent bike, per standard of care.

^b^ Used a body weight–adjusted treadmill.

^c^ Used patterned electrical neuromuscular stimulation.

**Table 2.** TKA and Outpatient Information

Characteristic	No. (%)			
	Control (n = 92)^a^	Intervention		
		Group 1 (n = 91)^b^	Group 2 (n = 90)^c^	Group 3 (n = 90)^b^^,^^c^
TKA information				
Performed at study’s host health system	67 (72.8)	64 (70.3)	74 (82.2)	62 (68.9)
Performed on left knee	60 (65.2)	40 (44.0)	41 (45.6)	44 (48.9)
Post-TKA length of stay, median (IQR), d^d^	3 (2-3)	3 (2-3)	3 (2-3)	2 (2-3)
Indication				
Wear and pain	75 (81.5)	71 (78.9)	78 (86.7)	72 (80.0)
Pain	3 (3.3)	4 (4.4)	0	2 (2.2)
Wear or tear due to OA	14 (15.2)	15 (16.7)	12 (13.3)	16 (17.8)
Previous TKA	21 (22.8)	14 (15.4)	14 (15.6)	14 (15.6)
Previous THA	4 (4.3)	2 (2.2)	5 (5.6)	3 (3.3)
Received other rehabilitation prior to outpatient PT	67 (72.8)	60 (65.9)	64 (71.1)	62 (68.9)
Received home health	60 (89.6)	45 (75.0)	57 (90.5)	55 (88.7)
Outpatient initial evaluation				
Time from TKA date to outpatient PT initiation, median (IQR), d^d^	20 (10-22)	18 (9-21)	20 (14-21)	19 (7-21)
Weight bearing status				
Full	30 (32.6)	27 (29.7)	29 (32.2)	21 (23.3)
As tolerated^e^	62 (67.4)	64 (70.3)	61 (67.8)	69 (76.7)
Used assistive device^e^	84 (91.3)	82 (90.1)	82 (91.1)	83 (92.2)
Pain score, mean (SD)^f^	4.5 (2.3)	4.4 (2.5)	4.7 (2.3)	4.7 (2.3)
Pain medications	88 (96.7)	83 (91.2)	85 (96.6)	84 (94.4)
Narcotic	76 (82.6)	69 (75.8)	76 (84.4)	76 (84.4)
OTC	31 (33.7)	28 (30.8)	26 (28.9)	29 (32.2)
Outpatient discharge evaluation				
PT course, median (IQR), d^d^	56 (39.5-73.5)	58 (45.5-73.8)	60.5 (43.2-75)	55 (44.8-74)
PT visits, mean (SD), No.	14.8 (5.7)	15.6 (5.5)	14.6 (6.2)	14.3 (6.3)
Full weight bearing	83 (90.2)	85 (93.4)	78 (86.7)	84 (93.3)
Used assistive device	9 (9.8)	6 (6.6)	15 (16.9)	9 (10.0)
Pain medications	54 (58.7)	49 (54.4)	52 (57.8)	49 (54.4)
Narcotic	24 (26.1)	16 (17.6)	38 (42.2)	15 (16.7)
OTC	40 (43.5)	38 (41.8)	39 (43.3)	38 (42.2)
Living at home	92 (100)	90 (98.9)	90 (100)	90 (100)

Abbreviations: IQR, interquartile range; OA, osteoarthritis; OTC, over the counter; PT, physical therapy; THA, total hip arthroplasty; TKA, total knee arthroplasty.

^a^ Used a recumbent bike, per standard of care.

^b^ Used a body weight–adjusted treadmill.

^c^ Used patterned electrical neuromuscular stimulation.

^d^ Values were computed using nonparametric Kruskal-Wallis test.

^e^ Weight bearing as tolerated indicates patients were cleared to place as much body weight as they could tolerate on the surgical leg and may have required use of an assistive device to do so. Using an assistive device does not indicate patients were not full weight bearing; this could be a safety precaution owing to balance issues or weakness or fatigue causing a patient’s surgical knee to buckle if they were not using one. Weight bearing as tolerated indicates that patients were cleared for 100% weight bearing but may have required gradual transition from partial to full weight bearing safely.

^f^ Range, 0 to 10, with higher scores indicating more pain.

Many patients received some form of post-TKA rehabilitation, mostly through home health, prior to their outpatient PT. The median (interquartile range) durations between patients’ TKA and initial outpatient evaluation were 20 (10-22) days in the control group, 18 (9-21) days in intervention group 1, 20 (14-21) days in intervention group 2, and 19 (7-21) days in intervention group 3. Each group had a similar level of functional mobility as measured by AM-PAC scores and by the 6-minute walk test at baseline (Table 3).

**Table 3.** Outcome Information Across Study Groups

Outcome measure	No. (%)				*P* value
	Control (n = 92)^a^	Intervention			
		Group 1 (n = 91)^b^	Group 2 (n = 90)^c^	Group 3 (n = 90)^b^^,^^c^	
At outpatient initial evaluation					
AM-PAC, mean (SD) [95% CI]	52.3 (6.2) [51.1-53.6]	51.2 (6.8) [49.8-52.6]	51.8 (5.8) [50.6-53.0]	51.7 (6.2) [50.5-53.0]	.67
34-51.9^d^	44 (47.8)	48 (52.7)	48 (53.3)	45 (50.0)	.52
52-65.9^e^	48 (52.2)	41 (45.1)	40 (44.4)	45 (50.0)	
66-83.9^f^	0	2 (2.2)	2 (2.2)	0	
6-min walk test, mean (SD) [95% CI], m	248.5 (91.6) [229.8-267.4]	241.5 (101.2) [220.6-262.4]	224.2 (89.7) [205.5-242.9]	226.2 (96.4) [206.1-246.3]	.25
At discharge from outpatient PT					
AM-PAC, mean (SD) [95% CI]	61.3 (5.3) [60.2-62.4]	61.3 (5.4) [60.2-62.4]	61.1 (6.1) [59.8-62.4]	61.2 (6.4) [59.9-62.5]	.99
34-51.9^d^	3 (3.3)	5 (5.5)	7 (7.8)	7 (7.8)	.44
52-65.9^e^	79 (85.9)	72 (79.1)	66 (73.3)	66 (73.3)	
66-83.9^f^	10 (10.9)	14 (15.4)	17 (18.9)	17 (18.9)	
6-min walking test, mean (SD) [95% CI], m	404.5 (107.8) [382.5-426.5]	385.7 (127.8) [359.3-412.1]	382.9 (128.6) [356.1-409.7]	395.2 (105.1) [373.3-417.1]	.60
Improvement from initial evaluation to discharge, mean (SD) [95% CI]					
AM-PAC	9.0 (8.4) [7.3-10.7]	10.0 (7.3) [8.5-11.5]	9.3 (6.4) [8.0-10.6]	9.4 (7.2) [7.9-10.9]	.80
6-min walking test, m	155.6 (95.9) [135.8-175.4]	144.2 (112.9) [120.9-167.5]	159.1 (125.2) [132.5-184.9]	168.4 (116.7) [144.1-192.7]	.50

Abbreviation: AM-PAC, Activity Measure for Post Acute Care.

^a^ Used a recumbent bike, per standard of care.

^b^ Used a body weight–adjusted treadmill.

^c^ Used patterned electrical neuromuscular stimulation.

^d^ Indicates limited mobility indoors.

^e^ Indicates moving around indoors.

^f^ Indicates moving around outdoors.

Patients were treated by a total of 21 licensed physical therapists who specialized in orthopedic and musculoskeletal rehabilitation across 15 outpatient clinics. Physical therapists enrolled and treated 10 to 68 patients each.

### Outcomes and Estimation

#### Discharge From Outpatient PT 

Outpatient treatment courses lasted approximately 60 days, including approximately 14 to 15 visits across 4 groups (Table 2). On discharge, most patients were able to bear their full weight without an assistive device (330 patients [90.9%]). More than half of study patients still used pain medications (204 patients [56.2%]), mainly over-the-counter medications as needed. Almost all lived at home on discharge from outpatient rehabilitation (362 patients [99.7%]).

#### Functional Outcomes 

Across groups, mean (SD) AM-PAC scores on discharge were similar (control: 61.3 [5.3]; intervention group 1: 61.3 [5.4]; intervention group 2: 61.1 [6.1]; intervention group 3: 61.2 [6.4]; *P* = .99). From baseline to discharge from outpatient rehabilitation, patients’ AM-PAC scores improved across groups (mean [SD] change: control, 9.0 [8.4]; intervention group 1, 10.0 [7.3]; intervention group 2, 9.3 [6.4]; intervention group 3: 9.4 [7.2]; *P* = .80).

On discharge, there was no statistically significant difference in mean (SD) distance patients from each group were able to ambulate based on the 6-minute walk test (control: 404.5 [107.8] m; intervention group 1: 385.7 [127.8] m; intervention group 2: 382.9 [128.6]; intervention group 3: 395.2 [105.1] m; *P* = .60). From baseline to discharge from outpatient therapy, patients were able to walk at least an additional 144.2 m across groups (mean [SD] change: control group, 155.6 [95.9] m; intervention group 1, 144.2 [112.9] m; intervention group 2, 159.1 [125.2] m; intervention group 3, 168.4 [116.7] m; *P* = .55).

Linear mixed modeling adjusting for patient demographic characteristics and differences in outpatient therapy duration and number of visits confirmed the unadjusted observations (eTable 1 in Supplement 2). Patients in the intervention groups did not have significantly improved AM-PAC scores or 6-minute walk test results at discharge compared with the change in the control group. Similarly, repeated measures linear mixed models did not show a statistically significant trend (eFigure 1 and eFigure 2 in Supplement 2).

## Discussion

This randomized clinical trial found no statistically significant differences in mobility outcomes among the control group and all 3 intervention groups as measured by AM-PAC scores. Patients achieved a similar functional level at discharge and progressed from baseline to discharge more than 2-fold (ie, ≥9.0 points) the suggested minimally detectable clinical change of 4 points.^39,40,41,42^ More than 92% of study patients were able to either move around indoors (AM-PAC score, 52-65.9) or outdoors (AM-PAC score, 66-83.9) on discharge, a substantial improvement from almost half of patients with limited mobility indoors (AM-PAC score, 34-51.9) at baseline. We also found no statistically significant differences in patient performance on the 6-minute walk test. Patients were able to walk at least an additional 144.2 m, more than 2-fold the minimal detectable change of 61.3 m suggested by Kennedy et al.^48^

This randomized clinical trial yielded results similar to previous outpatient-based studies^16,21^ in which patients improved their post-TKA functional community mobility following the outpatient rehabilitation at a similar rate, regardless of study group. It may be possible that the lack of significant differences among the control and intervention groups is, in part, mediated by the prolonged period prior to the initiation of outpatient PT (ie, 15-16 days after TKA). The optimal window, if any, to incorporate either the body weight–adjustable treadmill or a PENS unit to address key barriers (fear of falling and post-TKA pain) may have subsided by the time some study patients started outpatient therapy.

### Clinical and Policy Implications and Implications for Future Research

There is a wide array of different post-TKA therapy modalities, many claiming to improve outcomes while decreasing the rehabilitation time needed. Smaller clinics struggle to afford the cost or find the space needed for cutting-edge equipment that promise accelerated results. This study showed that recovery was similar regardless of the type of equipment used. Further study is needed to determine whether specific subpopulations would benefit differentially from each of the modalities used in this study^65,66,67^ and whether there are differing characteristics among those who benefited most or least in a given study group. In addition, research is needed to determine if any better timing exists as to when (eg, earlier) incorporation of different modalities, such as those used in the study, would facilitate a faster recovery and yield better rehabilitation outcomes after TKA. eTable 2 in Supplement 2 presenting outcomes between early and later starters (a dichotomous divide) within each intervention may provide information to spur investigation on whether the timing of the interventions resulted in any clinically meaningful differences across groups.

Postacute care across all settings accounts for 73% of the variation in Medicare spending per beneficiary,^68^ with outpatient care being among the least expensive. However, there still remains untold variation in outpatient rehabilitation practice and costs. Given that TKA has become a relatively routine procedure with predictable cost trajectories, it behooves clinical leaders and health policy experts to identify fiscally responsible trajectories and protocols that produce optimal outcomes. This study’s findings of no clinically or statistically significant differences across 4 arms suggest that an important next step is to identify the most cost-effective protocol that will provide the best functional outcomes for this increasing population.

### Limitations

This study has some limitations. The study was confined to a single health system, which may limit the study’s generalizability but helped facilitate close collaboration with, and buy-in from, front-line clinicians and ensure consistent study protocol implementation and administration. Clinician participation added immense value to the study. It also speaks to the benefits of embedding a research study within practice settings where the evidence can be more quickly implemented, when applicable.^69^ Most importantly it may foster stakeholder ownership of results necessary for clinical practice change when study results warrant.

Lack of blinding owing to its infeasibility may have introduced unknown or unintentional bias during the course of the study. The generalizability of the study findings may be limited because it took place within a single health system with practice patterns that may not represent that of the other outpatient clinics.

## Conclusions

The study found no statistically or clinically significant differences across the four groups. Because outcomes were similar among groups, clinicians may instead consider relative cost in tailoring TKA rehabilitation.

## Notes

Supplement 1. **Trial Protocol**

Supplement 2. **eTable 1.** AM-PAC and 6-Minute Walk Test Linear Mixed Model Coefficients and Interactions **eFigure 1.** AM-PAC Scores Over Time **eFigure 2.** 6-Minute Walk Test Performances Over Time **eTable 2.** AM-PAC and 6-Minute Walk Test Scores Between Early and Later Starters

Supplement 3. **Data Sharing Statement**
