# Supplementary material for: Effect of Outpatient Rehabilitation on Functional Mobility After Single Total Knee Arthroplasty: A Randomized Clinical Trial
Source: JAMA Netw Open. 2020 Sep 17;3(9):e2016571. doi: 10.1001/jamanetworkopen.2020.16571 (PMC7499127; doi:10.1001/jamanetworkopen.2020.16571)
Supplement: Supplement 1. — Trial Protocol [file jamanetwopen-e2016571-s001.pdf]

Knee Arthroplasty Rehabilitation Outcomes Study (KAROS)

INTRODUCTION

The number of people who undergo a total knee arthroplasty (TKA) procedure has increased rapidly in recent decades. The result is a function of increases in both demand and supply. On the demand side is a growing older population that is also experiencing increased longevity with increased likelihood of osteoarthritis. Moreover, the rising prevalence of obesity increases the probability of developing osteoarthritis at a younger age. On the supply side, there is an increase in numbers of orthopedic surgeons doing knee replacements and advances in medical technology, e.g., less invasive surgeries, are also associated with growing number of TKAs.[1]

Combined these trends have increased the demand for post-acute rehabilitation services to help individuals restore their physical function post-surgery. Outpatient physical therapy provides effective rehabilitation for individuals with TKA often in conjunction with other modalities, such as aquatic exercise, neuromuscular stimulation, and treadmill training. However, there is increased pressure for providers to search for a more robust rehabilitation program that can accelerate functional recovery, especially functional community mobility, among patients with a total knee arthroplasty. In the proposed study, we define functional community mobility as community ambulation, using alternating gait up and down 2 sets of stairs (10-14 steps/set) using one handrail, and transferring in and out of cars/chairs without assistance..[2,3,4,5,6,7,8,9,10,11]

Early and more intensive rehabilitation therapy has been shown to be associated with better functional outcome post TKA.[12,13,14] This often requires active and full-body engagement, e.g., being weight bearing, having an active range of motion, participating in gait training. However, two barriers keep TKA patients from being fully engaged in early and intensive physical therapy: (1) fear of falling, and (2) post-surgical pain especially when weight bearing. These barriers can prolong the recovery period and delay return to community or work.

Previous studies have shown that supporting a portion of a patient's body weight during gait training can help facilitate a patient's voluntary involvement in more aggressive therapy, in part, by mitigating pain resulting from body weight. Similarly, studies using harnessed body-weight support systems have also showed positive associations with functional gain.[15,16,17,18,19] Many body-weight support systems come with several drawbacks such as the extra time spent on preparing for patients to be properly and safely harnessed and the discomfort caused by strapping. In contrast, a FDA cleared anti-gravity treadmill® developed from patented NASA technology has become available. It supports or unloads a proportion of body weight during therapy but without any harness or straps. In addition, the technology provides precise partial weight bearing by accurate implementation of unweighting protocols that allow therapists to track and monitor patient's progress objectively over time. Studies using the anti-gravity body weight-supported treadmill have shown positive impact on rehabilitation outcomes among children with cerebral palsy and patients undergoing knee surgery and operation of Achilles tendon.[20,21,22]

In addition, modalities, such as electrical stimulation, have been used to complement conventional physical therapy. Research has demonstrated the effectiveness of electrical stimulation for neuromuscular re-education following surgery.[23,24,25,26,27] Neuromuscular stimulation has been used safely for a long time to help manage pain, relieve muscle spasms, increase range of motion, prevent muscle disuse atrophy, increase circulation and most

importantly, re-educate muscles. Recently, a state-of-the-art neuromuscular stimulation device became available using a patterned waveform. This device is thought to better facilitate neuromuscular re-education because its patterned stimulation pattern mimics the firing pattern of muscles during ballistic, reciprocal, or functional patterns.

OBJECTIVES

The proposed study has three objectives:

1. Compare rehabilitation outcomes of using an anti-gravy treadmill (known as AlterG) vs. a conventional physical therapy protocol among patients with a total knee arthroplasty;

2. Compare rehabilitation outcomes of using PENS-OMNISTIM® FX² Pro Electrotherapy System (hereafter, PENS) vs. a conventional physical therapy protocol among patients with a total knee arthroplasty;

3. Compare rehabilitation outcomes of using both AlterG and a patterned electrical neuromuscular stimulation (PENS; OMNISTIM® FX² Pro Electrotherapy System) (hereafter, AlterG^{+}) vs. a conventional physical therapy protocol among patients with a total knee arthroplasty.

Hypotheses:

1. We hypothesize that there are near-term clinically significant rehabilitation outcome differences in functional mobility following total knee arthroplasty between (a) patients in the control group who receive a conventional outpatient physical therapy and (b) patients in the AlterG group who receive anti-gravity treadmill therapy.

2. We hypothesize that there are near-term clinically significant rehabilitation outcome differences in functional mobility following total knee arthroplasty between (a) patients in the control group who receive a conventional outpatient physical therapy and (b) patients in the PENS group who receive therapy using the OMNISTIM® FX² Pro system.

3. We hypothesize that there are near-term clinically significant rehabilitation outcome differences in functional mobility following total knee arthroplasty between (a) patients in the control group who receive a conventional outpatient physical therapy and (b) patients in the AlterG^{+} group who receive a combination of anti-gravity treadmill and PENS.

DESCRIPTION OF DEVICES

The AlterG Anti-Gravity Treadmill® (AlterG)

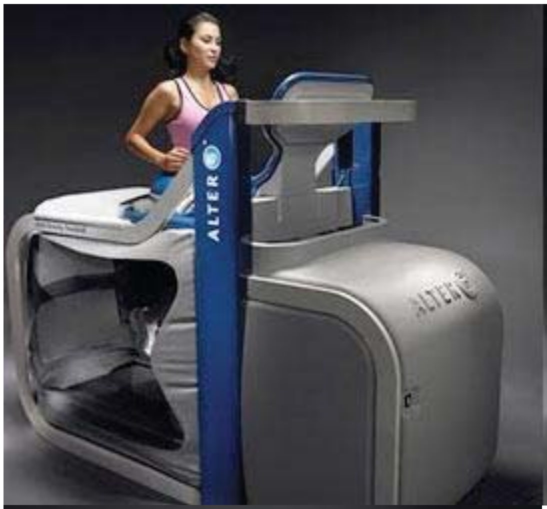

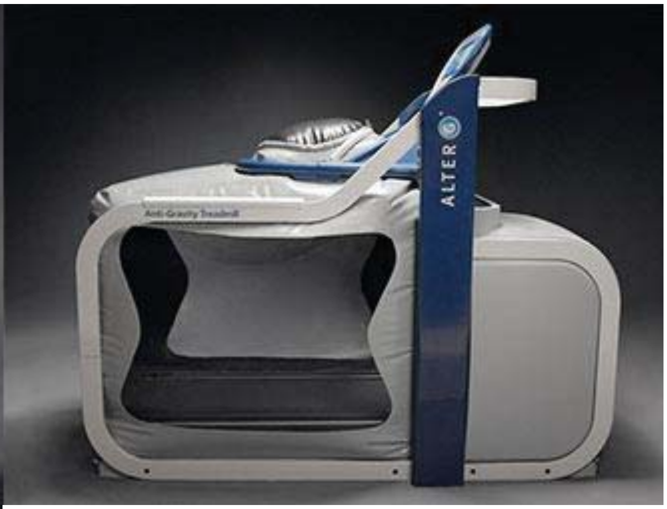

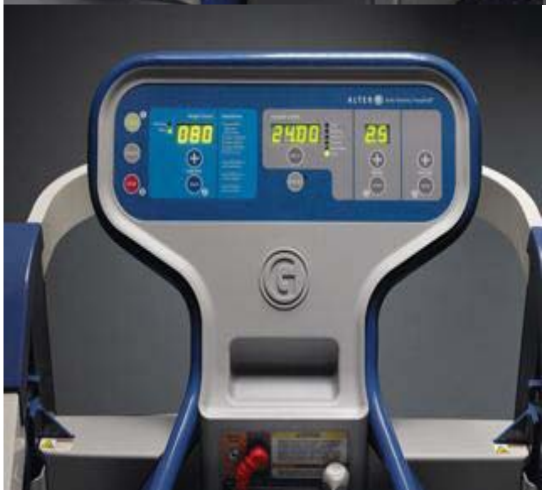

AlterG, cleared by the FDA in 2008 (see Appendix A-1 for FDA clearance letter), is an anti-gravity treadmill that uses different air pressures to unload or support a proportion of body weight during therapy. The concept of using differential air pressure to support body weight was originally conceived by Dr. Robert Whalen and his team when they designed an exercise program for NASA astronauts. Initially, Dr. Whalen and team proposed to use differential air pressure in space to add weight to the body to mimic the earth's gravity during exercise to prevent bone loss and muscle atrophy among astronauts. AlterG evolved from Dr. Whalen's original idea using air pressure for the opposite effect, namely lifting weight from the body during training or therapy to allow for protection of healing tissue, facilitate full range of motion and early initiation of closed-kinetic chain activity, prevent disuse muscle atrophy, and encourage more rapid return of motor control.

AlterG allows a patient to be mobile at a fraction of his/her body weight. It helps the therapist identify where (i.e., at what proportion of body weight support) the pain stops and patient can move freely. This, in turn, not only promotes the rate of muscle conditioning, but also

facilitates early initiation of closed kinetic chain activity. These prerequisites help prepare patient for more intensive subsequent one-on-one therapeutic exercise regimen. AlterG proposes that it will facilitate patient's return to community ambulation more quickly than standard protocol due to decreased fear of fall, weight and pain while gait training.

AlterG includes the following components: a base frame, a treadmill, an upper support frame, the inflatable bag, a pressure source and regulation system, control electronics and a control console. The treadmill used in the AlterG is equivalent to that used in predicate (existing) devices. They all allow for the adjustment of walking/running speed with similar gradation mechanism. The AlterG has an additional structure that surrounds the patient in the event of fall. The unloading mechanism used in the AlterG differs technologically that it uses a pressurized bag to provide a counterforce to an individual's body weight, reducing their effective weight on the treadmill surface. The therapist can adjust the patient's effective weight bearing on the treadmill surface by adjusting the pressure in the inflatable bag. See Appendix A-2 for instructional manual.

OMNISTIM® FX² Pro Electrotherapy System (PENS)

Developed by ACP, OMNISTIM® FX² Pro Electrotherapy System features a unique electrical stimulation pattern, namely, Patterned Electrical Neuromuscular Stimulation (PENS), that closely replicates the body's normal muscle and nerve firing patterns (agonist and antagonist or reciprocal muscle pairs) in triphasic, biphasic, or functional patterns. See Appendix B-1 for FDA clearance and approval. This neuromuscular re-education approach provides a high intensity and accurately timed sensory input, which mimics the firing pattern of sensory neurons and muscle stretch during voluntary activity. PENS at its patented stimulation pattern will increase proper muscle recruitment and thereby facilitate muscle strengthening more effectively than usual care. The model offers protocol-use recording capabilities, a built-in lead wire/output tester, and evidence-based condition-driven operational protocols for ease of use. The system also includes a control feature that allows therapists to provide active-assisted stimulation for functional exercises.

The OMNISTIM® FX² Pro's two separate generators – Medium Frequency Alternating Currents (MFAC) and Low Voltage Pulsed Current (LVPC) – produce medium frequency (2000, 2500, 4000, 5000 or 10,000 Hz) alternating current in continuous or modulated modes or asymmetric biphasic pulsed current. The OMNISTIM® FX² Pro's MFAC and LVPC modes come with fully adjustable ON and OFF Times and ON and OFF Ramps which provide a wide variety of uses for muscle re-education and muscle spasm reduction programs for innervated muscle. Two isolated output circuits with independent intensity controls are provided. The output of each circuit is easily determined in milliamps through the display screen. The digital timer allows the operator to select the length of the total treatment time and to monitor the time remaining in minutes. (See Appendix B-2 for instructional manual.)

RESEARCH STRATEGY

Study Design

The proposed study is a randomized, partially blinded, four-arm parallel controlled trial (see Intervention Section for detailed description of treatment protocols for each group):

Arm 1: Standard of care -- Conventional post-surgical TKA therapy (Control group)
Arm 2: AlterG
Arm 3: PENS -- OMNISTIM® FX^2 Pro Electrotherapy System
Arm 4: AlterG^{+} -- Alter-G and PENS OMNISTIM® FX^2 Pro Electrotherapy System

We considered a 2x2 factorial study design which can potentially reduce the sample size needed. But, we determined that a 2x2 factorial design may not be appropriate because of the underlying assumption that two study interventions, in this case, AlterG and PENS, must act independently. That is, the assumption dictates that AlterG is equally effective whether or not the patient is receiving PENS, and vice versa, and the two treatments do not have a potential for substantial interaction.[28] We think that the possibility likely exists that AlterG and PENS may have an interaction effect when being used concurrently. Furthermore, the resulting smaller sample size using a 2x2 factorial design may lead to inconclusive findings which usually cannot be overcome by analytic strategies. With limited evidence to guide us, we decided to use a four-arm parallel trial design.

Study Facilities:

A total of 15 outpatient settings from the MedStar National Rehabilitation Network across Baltimore-DC region will participate in the proposed study:

MedStar NRH Rehabilitation Network	Location	[redacted]
NRH Outpatient Center for Orthopedic Rehabilitation (OCOR)	102 Irving St. NW, Washington, DC	
NRH Rehabilitation Network, Oxon Hill	6196 Oxon Hill Rd., Suite 450, Oxon Hill, MD	
NRH Rehabilitation Network, Olney	18109 Prince Philip Drive, Suite 155, Olney, MD	
NRH Rehabilitation Network, Friendship Heights	5530 Wisconsin Ave. Suite 960, Chevy Chase, MD	

NRH Rehabilitation Network, Sports Medicine	1407 York Road, Suite 100, Lutherville, MD	[redacted]
NRH Rehabilitation Network, Harbor Hospital Sports Medicine	2900 South Hanover St. Suite 102, Baltimore, MD	[redacted]
NRH Rehabilitation Network, Bel Air	658 Boulton St. Suite A, Bel Air, MD	[redacted]
NRH Rehabilitation Network, Perry Hall	5009 Honeygo Center Dr. Suite 209, Perry Hall, MD	[redacted]
NRH Rehabilitation Network, Bethesda	6410 Rockledge Dr. Suite 600, Bethesda, MD	[redacted]
NRH Rehabilitation Network, Stadium Place	900 East 33rd St., Baltimore, MD	[redacted]
NRH Rehabilitation Network, Salisbury	1655 Woodbrooke Dr., Suite 102, Salisbury, MD	[redacted]
NRH Rehabilitation Network, Ellicott City	Dorsey Hall Medical Center, 9501 Old Annapolis Rd., Ellicott City, MD	[redacted]
NRH Rehabilitation Network, McLean, VA	6858 Old Dominion Dr. Suite 200, McLean, VA	[redacted]
NRH Rehabilitation Network, Westminster, MD	412 Malcolm Dr. Suite 200, Westminster, MD 21157	[redacted]
Union Memorial Hospital Rehabilitation & Sports Medicine	3333 North Calvert St., Suite 300, Baltimore, MD 21218	[redacted]

Study Group

Inclusion criteria: Patients who fulfill the following selection criteria will be eligible for the study.	
1	Patients who undergo an *elective unilateral* total knee arthroplasty and initiate their outpatient physical therapy within 24 days post-TKA procedure.
2	Patients who are 40 years old or older.
3	Patients who weigh less than 300 lb[1].
4	Patients who underwent any lower extremity joint replacement (including total hip arthroplasty, or THA) ≥ 1 year prior the current TKA procedure and fulfill all selection and exclusion criteria for the study, are eligible for enrollment if they successfully completed their rehabilitation program for their previous procedure.
Exclusion criteria: Patients who have any of the following conditions will be excluded from the study.	
1	Patients who undergo any lower extremity joint replacement procedure, including a revision, or second, or bilateral TKA, and THA, ≤ 1 year prior the current TKA.
2	Patients whose payer (primary or secondary) is workers' compensation.
3	Patients who are in litigation related to injury or disease associated with their current TKA.
4	Patients who are younger than 40 years old.
5	Female patients who are pregnant or may be pregnant.
6	Patients who weigh 300 lbs or more.
7	Patients who have a medical history of neurologic disorders (e.g., multiple sclerosis, Parkinson's, stroke), rheumatoid arthritis, or gout (unless ≥6 months since last exacerbation/flare up and under control medically, e.g., medications).[2]
8	Patients who are under active cancer treatment (chemotherapy, radiation), with history of malignancy in either of both lower extremities, or with evidence of signs/symptoms of cancer, chemotherapy or radiation less than one year prior current TKA.
9	Patients who develop deep vein thrombosis.
10	Patients who undergo a TKA due to traumatic injury.
11	Patients who are unable to proceed or continue the planned outpatient program, because of complications such as wound infection related to the TKA, severe orthostatic hypotension,[3] and manipulation.[4]
12	Patients who have received more than 2 weeks of post-TKA home health care or other post-acute services prior to outpatient physical therapy.

[1] To accommodate the weight limit of 320 lb to use Alter-G.
[2] Patients with peripheral neuropathy are eligible for the study if they fulfill all other criteria.
[3] For its contraindication for exercise.
[4] This subset of patients requires a manipulation procedure due to limited movement post-TKA. This subset of patients usually undergoes a different therapy protocol designed specifically for post-manipulation. We will exclude this subset of patients.

13	Patients who have cardiac demand pacemakers and/or implanted defibrillators.[5]
14	Patients who have uncontrolled cardiovascular hypertension.

Interventions

The proposed study is a 4-arm randomized controlled trial to compare the rehabilitation outcomes between three intervention groups and the control group for total knee arthroplasty. The intervention protocols for each of the 4 groups are described as follows (also see Table 1):

Control group (Arm 1)

Study patients in the control group will receive a conventional 12-week long physical therapy program designed for TKA. Patients will receive 2-3 treatment sessions per week during the first two weeks, followed by 2 sessions per week for 6 weeks, and 1 session per week during the last month of the program. During the very first session in Week 1 (Visit 1), physical therapists will perform a comprehensive initial evaluation for each study patient, therefore, patients will not receive treatment during the first session.[6] Each treatment session following the initial evaluation lasts for approximately 70minutes, starting from a therapeutic exercise/warm-up regimen, followed by a course of therapeutic activity (treatment phase), and a subsequent cool-down phase. ***The therapeutic exercise*** regime refers to exercises designed by physical therapists to work on patient physiological body exertion, to improve body function, or to improve or restore strength, endurance, flexibility, range of motion, or coordination. The therapeutic exercise aims to condition and prepare patients for subsequent functional or therapeutic activities and is routinely done in conjunction with modalities, such as Nu-step or recumbent bike. ***The therapeutic activity*** (treatment phase) comprises a repertoire of activities that address strengthening and neuromuscular re-education and includes manual therapy. Designed by physical therapists, the therapeutic activity program is usually tailored to individual patient needs and functional goals. The therapeutic activity program is typically a one-on-one format working directly with a physical therapist. Physical therapists devise a personalized treatment plan and provide specific individual instructions on how to appropriately perform or carry out therapeutic activities. ***The cool-down down*** phase allows the body to gradually transition from an exertion state to a resting state after an intensive therapy session. Physical therapists will also provide pain and inflammation management at this phase to conclude the treatment session.

AlterG group (Arm 2)

Study patients in the AlterG group will receive a comparable 12-week long physical therapy program. Each treatment session will be the same length as the control group. They will perform the ***therapeutic exercise*** regimen using AlterG by unloading a proportion of their body weight to facilitate conditioning and prepare for subsequent therapeutic activities. Specifically,

[5] Per contraindications for Omnistim FX^2 Pro.
[6] This is a standard practice across MedStar outpatient therapy centers. All patients undergo a comprehensive evaluation at their first outpatient visit allowing therapists to gather necessary information to devise treatment goals and plan.

physical therapists will identify the threshold, in terms of body weight unloading, that minimizes pain and allows patients to feel comfortable to move freely while on AlterG. In addition to partial unweighting, physical therapists will also determine the appropriate walking speed for each patient while on AlterG. Study patients in the AlterG group will then participate the same ***therapeutic activity*** regimen during the treatment phase as the control group mentioned above, except that patients in this group will perform select closed chain activities using AlterG in static mode, for example, heel raises, high march steps, and single leg stance activities. Patients in the AlterG group will perform the same ***cool-down*** regimen as the control group. Physical therapists will use the Progression Log table (see Tables 2a) to document AlterG parameters, that is, % of body weight unloading, speed, and time on AlterG, for each patient at each treatment session as well as the pain score.

PENS group (Arm 3)

Study patients in the PENS group will receive a comparable 12-week long physical therapy program. Each treatment session will be the same length as the control group. They will perform the ***therapeutic exercise*** regimen using PENS. Specifically, during the first 3 weeks, patients will use the LE Triphasic program for 10 minutes, followed by another 10 minutes using LE Cycle program while on Nu-step or recumbent bike. After 3 weeks, patients should progress and will use LE Cycle program while on the bike for 15-20 minutes. Physical therapists will identify and record the setting of PENS used in each therapy session. Study patients in the PENS group will then participate in the same ***therapeutic activity*** regimen during the treatment phase as the control group mentioned above. Patients in the PENS group will perform the same ***cool-down*** regimen as the control group. Physical therapists will use the Progression Log Table 2b to document PENS parameters as well as pain score.

AlterG$^+$ group (Arm 4)

Study patients in the AlterG$^+$ group will receive a comparable 12-week physical therapy program with the same length of each treatment session as the control group. Patients in this group will use both AlterG and PENS during the ***therapeutic exercise*** phase. Specifically, during the first 3 weeks, patients will begin with PENS LE Triphasic pattern for 10 minutes, followed by PENS LE Walk program while walking on the AlterG for 10 minutes. After 3 weeks, patients will use AlterG and PENS LE Walk program simultaneously for 15-20 minutes. The combination will not only unload a proportion of patient's body weight (AlterG) but also facilitate muscle recruiting through the patented stimulation pattern in PENS during therapeutic exercises. The AlterG$^+$ group will then use the same one-on-one ***therapeutic activity*** program as the AlterG group. The AlterG$^+$ group will also implement the same ***cool-down*** regimen as those in the other two groups. Similarly, physical therapists will use the Progression Log tables to document AlterG parameters (Table 2a), PENS parameters (Table 2c), and the pain score for each patient at each treatment session.

Standardization of interventions

Interventions in each of the 4 arms are standardized to achieve consistent implementation of the intervention across participating sites. The study team will provide a comprehensive

training using a train-the-trainer approach to assure that site coordinators and treating physical therapists have a thorough and solid understanding in the following critical components: (1) knowledge of overall study design and objective, (2) knowledge of study protocol, (3) participant safety and monitoring, (4) equipment use (namely, how to operate the AlterG and PENS accordingly), and (5) standardized data collection protocol.

Table 1. Interventions —4-arm study

**Group/protocol (24 sessions)**	**Arm 1 Control (current)**	**Arm 2 AlterG**	**Arm 3 PENS only**	**Arm 4 AlterG^{+} (AlterG & PENS)**
**Phase 1: Therapeutic exercise/warm-up (15-20 minutes)**	Patients will perform therapeutic exercise using either recumbent bike or Nu-step.	Patients will perform therapeutic exercise using Alter-G in functional mode.	First 3 weeks: Patients will use LE Triphasic program for 10 minutes, followed by LE Cycle program for another 10 minutes.<br>After 3 weeks: Patients will use LE Cycle program while on a bike for 15-20 minutes.	First 3 weeks: Patients will use LE Triphasic program for 10 minutes, followed by LE Walk program while on AlterG-functional mode for another 10 minutes.<br>After 3 weeks: Patients will use PENS LE Walk program with AlterG-functional mode for 15-20 minutes.
**Phase 2: Treatment: One-on-One format (35-40 minutes)**	Patients will engage in therapeutic activities (including closed chain activities) that address the following:<br>• Strengthening – e.g., sidestepping, straight leg raise.<br>• Neuromuscular re-ed – e.g., single leg stance activities, hip flexion/extension/ adduction/ abduction.<br>• Manual therapy – e.g., stretching/ROM, scar tissue massage.	Patients will engage in the same therapeutic activity regimen as control group except that they will perform select closed chain activities on AlterG in static mode, e.g., high march steps, heel raises, single leg stance activities.	Patients will engage in the same therapeutic activity regimen as control group.	Patients will engage in the same therapeutic activity regimen as control group except that they will perform select closed chain activities on AlterG in static mode, e.g., high march steps, heel raises, single leg stance activities.

**Phase 3: Cool-down (15 minutes)**	• Vasopneumatic compression (pain & inflammation management)	Same as control group	Same as control group	Same as control group
**Duration and intensity**				
First 2 weeks	70-minute session, 2-3x/wk			
Weeks 3-8	70-minute session, 2x/wk			
Weeks 9-12	70-minute session, 1x/wk			
Month 6	Follow-up via a phone survey to examine specifically, but not limited to:<br>• Study participants' symptoms and functional level<br>• Amount of time to resume activity level prior to TKA (sustainability of treatment effect of Alter-G, PENS, and Alter-G$^+$)<br>• AM-PAC & KOOS (see Measures and Instruments Section)			

Appendix C provides a complete list of treatment or therapeutic activities that PTs can choose to use in the treatment phase.

Note:

If a patient is ready to be discharged before the proposed 20-22 visits, therapists should plan their discharge accordingly. No additional visits are required. We will use the outcome data captured at the last visit as the end data point for the patient using the intent-to-treat assumption. Similarly, if a patient requires more than proposed 20 or 22 visits, therapists should continue to provide treatment until he/she is ready for discharge. We will use the outcome data from their 20th or 22nd visit as the end data point for the study. But we will ask coordinators and therapists to perform and document the outcome measures at patient's last visit.

Table 2a. Progression Log Table when using Alter-G during therapeutic exercise phase (functional mode) and therapeutic activity phase, closed chain activity only (static mode[7])

Weeks since outpatient PT	% body weight	Speed (MPH)	Time on Alter-G (minutes)	Distance	% body weight	Type of Closed Chain Activity	Time on Alter-G (minutes)	Pain score 0-10
Functional mode					Static mode			
Week 1								
Visit 1	Initial evaluation only							
Visit 2 or Visits 2-3								
Week 2 Visits 3-4 or Visits 4-6								
Week 3 Visits 5-6 or Visits 7-8								
Week 4								

[7] Treadmill turned off.

Visits 7-8 or Visits 9-10								
Week 5 Visits 9-10 or Visits 11-12								
Week 6 Visits 11-12 or Visits 13-14								
Week 7 Visits 13-14 or Visits 15-16								
Week 8 Visits 15-16 or Visits 17-18								
Week 9 Visit 17 or Visit 19								
Week 10 Visit 18 or Visit 20								
Week 11 Visit 19 or Visit 21								
Week 12 Visit 20 or Visit 22								

Table 2b. Progression Log Table when using PENS in Arm 3

Weeks since outpatient PT	OMNISTIM® FX² Pro-PENS	Time using PENS (minutes)	Pain score 0-10
Week 1	Intensity setting[8]		
Visit 1	Initial evaluation only		
Visits 2-3	LE Triphasic program		
	LE Cycle program		
Week 2	LE Triphasic program		
	LE Cycle program		
Week 3	LE Triphasic program		
	LE Cycle program		
Week 4	LE Cycle program		
Week 5	LE Cycle program		
Week 6	LE Cycle program		

[8] Therapist will select the baseline PENS setting for each individual based on the setting that induces firing of muscle/nerve and patient's comfort level (not painful). The intensity setting should diminish over time per patient's (muscle/nerve) response to stimulation.

Week 7	LE Cycle program		
Week 8	LE Cycle program		
Week 9	LE Cycle program		
Week 10	LE Cycle program		
Week 11	LE Cycle program		
Week 12	LE Cycle program		

Table 2c. Progression Log Table when using PENS in Arm 4

Weeks since outpatient PT	OMNISTIM® FX² Pro-PENS	Time using PENS (minutes)	Pain score 0-10
Week 1	Intensity setting		
Visit 1	Initial evaluation only		
Visits 2-3	LE Triphasic program		
	LE Walk program		
Week 2	LE Triphasic program		
	LE Walk program		
Week 3	LE Triphasic program		
	LE Walk program		
Week 4	LE Walk program		
Week 5	LE Walk program		
Week 6	LE Walk program		
Week 7	LE Walk program		
Week 8	LE Walk program		
Week 9	LE Walk program		
Week 10	LE Walk program		
Week 11	LE Walk program		
Week 12	LE Walk program		

Measures and Instruments

AM-PAC

AM-PAC (Activity Measure for Post Acute Care)[29,30] is a computerized adaptive testing instrument developed by Boston University Health and Disability Research Institute (BU HDRI) to measure functional level in 3 domains: basic mobility, daily activity, and applied cognition. Using item-response theory, AM-PAC program selects the most representative questions from its extensive item bank to ask a patient to measure their functional level according to a few simple indicators, such as age, gender and impairment. It usually takes only on average 6 to 7 questions in each domain to identify a patient's functional level. It takes less than 5 minutes to administer the AM-PAC. The program generates a report containing continuous scores for each domain tested. All scores are automatically stored in a Microsoft Access data table. For the purpose of the study, we will only implement two domains in the AM-PAC, including basic mobility and daily activity, as patients who undergo an elective TKA do not have any cognitive issue in general. We propose to use AM-PAC as the primary outcome measure for the study because of its robust and extensive validity. See Appendix D for an example list of questions from the item bank of the AM-PAC.

6-Minute Walk Test (6MWT)

The 6-minute walk test is a measure of functional capacity. It was initially developed to evaluate walking endurance among frail elderly patients 60-90 years of age.[31] The test since has been used as a performance-based measure of functional capacity in various populations, including healthy older adults, people with stroke, and patients undergoing knee or hip arthroplasty.[32,33,34,35,36,37,38,39,40,41,42] The 6MWT measures the distance an individual is able to walk over a duration of six minutes on a hard, flat surface. Patients are instructed to walk as far as possible in the fixed amount of time. They are allowed to use any assistive device during the test as well as rest as often as they need. The stopwatch will continue to count the time even while patient is resting. The distance the patient is able to walk, use of and type of assistive devices and number of rests within the six minutes will be documented for each study patient. See Appendix E for assessment instructions.

Walking Speed

Walking speed, which has been suggested as the sixth vital sign by physical therapy,[43] has been found to correlate with balance, functional ability, and future health status. [9,44,45,46,47] Progress in walking speed is also associated with clinical meaningful changes in quality of life and community mobility.[48,49] Walking speed will be tested over a 10-meter course on a level surface. Patients will ambulate on the testing course without any assistance at their comfortable usual speed. Time is measured for the intermediate 6 meters to allow for acceleration and deceleration and documented. Walking speed (m/s) is then calculated as 6 meters divided by the time (in seconds) it takes for a patient to walk. See Appendix F for assessment instructions.

The Knee Injury and Osteoarthritis Outcome Score (KOOS)[50]

The Knee injury and Osteoarthritis Outcome Score (KOOS) is a knee-specific instrument. It was developed as an extension of the well known WOMAC[51] Osteoarthritis Index with the purpose of including quality-of-life domain that the WOMAC lacked and covering both short-term and long-term outcomes. The KOOS includes 5 domains that measure (1) pain-9 items, (2)

other symptoms-7 items, (3) function in daily living (ADL)-17 items, (4) function in sport and recreation-5 items, and (5) knee-related quality of life-4 items. See Appendix G for the complete KOOS questionnaire. The KOOS has been validated for several orthopedic interventions, most notably, total knee replacement and for other physical therapy interventions, use of nutritional supplementation, and others.[52] The 5 subscales are scored separately using a Likert scale. Each item/question has five possible options scored from 0 being no problem to 4 being an extreme problem. Scores from individual item within each domain will be summed up to be the score of each domain. Scores will then be transformed to a 1-100 scale with 0 meaning extreme knee problems and 100 indicating no knee problems. Scoring algorism is available in Microsoft Excel format and can be downloaded from www.koos.nu.

Pain Score

This is a patient self-reported 11-point numeric rating scale from 0 to 10 asking patient to rate the level of pain at each treatment session. We propose to use the JCAHO approved pain assessment scales to measure study patient's pain per JCAHO (Joint Commission on Accreditation of Healthcare Organizations) pain management standards.[53] See Appendix H for MedStar National Rehabilitation Network Pain Management Policy and pain assessment tool. A score of 0 indicates no pain and a score of 10 indicates worst pain possible. Prior each therapy session, we will capture patient's pain level by asking how they rate their pain level now.

Follow-up Survey

We propose to conduct a phone interview at 6-month post-TKA to collect outcome data for study patients, for example, level of functional independence via AM-PAC and quality of life via KOOS. Other outcome data includes medical complications and downstream health care utilization, such as rehospitalization and ER visit posts-TKA, employment status or return to work if applicable, societal participation, and patient's overall satisfaction in terms of treatment program and rehabilitation outcomes. We propose to administer a telephone follow-up instrument akin to the one provided in Appendix I that was developed and used in the previous 22-site JOINTS (Joint Replacement Outcomes in Inpatient Rehabilitation Facilities and Nursing Treatment Sites) I & II studies.[12,54] We will modify this instrument and review the instrument with the Clinical Practice Team prior to pilot testing and actual implementation. We propose to develop a follow-up questionnaire, much shorter than the example provided in Appendix I, which will take approximately 10-15 minutes to administer. We will submit for an IRB amendment review once the instrument is finalized. We will not implement the follow-up survey until the IRB amendment approval is received.

Table 3. Measures and Instruments

Instrument	Measure	Frequency	Remarks
ROM	Range of motion	• Initial evaluation<br>• 10th visit[9]<br>• End of month 2<br>• End of month 3	Knee joint only; routine clinical assessment
Functional manual muscle testing	Muscle strength	• Initial evaluation<br>• 10th visit<br>• End of month 2<br>• End of month 3	Assessment done during treatment; routine clinical assessment
AM-PAC	Functional ability (basic mobility & daily activities)	• Initial evaluation<br>• 10th visit<br>• End of month 2<br>• End of month 3<br>• Follow-up	Web-based application
6-minute walk	Ambulation distance in a fixed duration	• Initial evaluation<br>• 10th visit<br>• End of month 2<br>• End of month 3	Performance-based
Timed 10-meter walk	Walking speed	• Initial evaluation<br>• 10th visit<br>• End of month 2<br>• End of month 3	Performance-based
KOOS	Short-term and long-term symptoms and function (pain, symptoms, function in ADL, function in sports and recreation, and QoL)	• Initial evaluation<br>• 10th visit<br>• End of month 2<br>• End of month 3<br>• Follow-up	Self-administered

[9] Or 30th day whichever comes first.

Sample Size Calculation

I. Primary Hypotheses

Hypothesis 1:
H0: $\mu 1=\mu 2$, where $\mu 1$ is the mean of change in the basic mobility domain of the AM-PAC from baseline to end of the 12-week outpatient program in control arm and $\mu 2$ is the mean of change in the basic mobility domain of the AM-PAC from baseline to end of the 12-week outpatient program in the AlterG arm.
H1: $\mu 1\neq\mu 2$

Hypothesis 2:
H0: $\mu 1=\mu 2$, where $\mu 1$ is the mean of change in the basic mobility domain of the AM-PAC from baseline to end of outpatient program in control arm and $\mu 2$ is the mean of change in the basic mobility domain of the AM-PAC from baseline to end of outpatient program in the PENS arm.
H1: $\mu 1\neq\mu 2$

Hypothesis 3:
H0: $\mu 1=\mu 2$, where $\mu 1$ is the mean of change in the basic mobility domain of the AM-PAC from baseline to end of outpatient program in control arm and $\mu 2$ is the mean of change in the basic mobility domain of the AM-PAC from baseline to end of outpatient program in the AlterG^{+} arm.
H1: $\mu 1\neq\mu 2$

II. Sample Size

We estimated the study power using the PASS (Power Analysis and Sample Size Software, Kaysville, UT) 2008. The primary outcome of this study is the change in the basic mobility domain of the AM-PAC from baseline to end of the 12-week outpatient program. The power calculations were based on primary hypotheses using Student t-test. We then adjusted the sample size using Wilcoxon rank-sum test relative to any statistical test. This adjusted sample size will ensure adequate power to detect the projected effect size with any underlying distribution. The power was set at 80%. The level of significance was set at 0.016, i.e., 0.05/3, with Bonferroni adjustment for multiplicity. Based on the result of previous study[29,30] and clinical judgment, we set the difference in means between two arms to 4 and the standard deviation (SD) to 5-10.

Table 4 presents sample size estimates based on an 80% power and the primary hypotheses aforementioned under different scenarios (i.e., SD, loss to follow-up rate).

Table 4. Power calculation based on primary hypothesis (**power=80%**, alpha=0.016)						
μ1-μ2	SD	N in each arm	# in each arm adjusted for loss to follow-up/dropouts		Total N (4 groups) adjusted for loss to follow-up/dropouts	
			20%	10%	20%	10%
4	10	140	175	156	700	624
4	9	113	142	126	568	504
**4**	**8**	**90**	**113**	**100**	**452**	**400**
4	7	69	87	77	348	308
4	6	51	64	57	256	228
4	5	36	45	40	180	160

Per recommendations from the AM-PAC development group led by Alan Jette, PT, PhD, we used a mean difference of 4 points with a standard deviation (SD) of 8[29,30] in the domains of Basic Mobility and Daily Activity in the AM-PAC as a clinically meaningful change to calculate sample size for the study. To achieve an 80% power, the minimal sample size required for each group is N=90. To account for a 20% potential sample attrition (patient drop-outs, inability to complete the study protocol), we propose a minimal sample size of N=113 for each of the 4 groups (total proposed N=452).

Randomization Method and Blinding

After an eligible study participant agrees and provides a signed informed consent, he/she will be assigned a study ID consisting of a 7-digit number where the first 3 digits represents site ID concatenated with a 3-digit consecutive run-in number representing participant's study ID. For example, the first participant entering the study site 003 will receive a study ID of 0031001. Randomization of study group will occur immediately after enrollment. Participants will be randomly assigned to one of the four treatment groups: control group, AlterG, PENS, or Alter G^+. We propose to use a randomized permuted block design of block size eight to assign participant to one of the four treatment groups. We will perform separate randomization for each study site. In each study site, there is a 1:1:1:1 random assignment of participants to the four treatment groups. We choose to stratify randomization based on study site to address the potential center effect or bias. These biases can stem from differences in patient populations, care management, and other contextual factors that are unique to each individual study site. The MHRI Department of Biostatistics and Epidemiology will provide instructions to each study site on how to utilize randomization scheme, a newly added feature in REDCap to perform participant's randomization. The inclusion/exclusion checklist will be incorporated into the randomization list to ensure all randomized participants are eligible.

It is not feasible to blind study participants and treating physical therapists about participant's treatment group assignment mainly because of the nature of the study. In addition, due to resource constraints, we will not be able to employ blinded evaluators to perform

outcomes measures. Alternatively, we propose to have all site coordinators who are less likely to be treating clinicians to perform outcome measures for each study participant at baseline, 10th visit or 30th day, end of month 2, and end of month 3. Within each study site, we will also have designated treating physical therapists implementing one of the four study protocols. For example, PT Smith will be implementing treatment protocol for AlterG arm. All participants who are randomized to AlterG will be under PT Smith's care. To mitigate potential selection bias caused by non-blindness, we have carefully chosen two objective measures with extensive and excellent inter-rater reliability as well as validity with little vulnerability to rater bias as our primary outcome measures, including AM-PAC and 6-minute walk test. See Measures and Instruments section. However, blinded raters will be used to obtain all follow-up data via phone interview. These raters will be supervised by investigators to minimize bias and patients will be instructed not to reveal their treatment assignment to these raters during the follow-up phone interview.

Statistical Analyses

Preliminary analysis: We will examine the descriptive statistics, frequency distribution and graphic plots of each variable to detect the data errors, outlying values, number and pattern of missing data and normality of distributions. The natural log transformation will be applied to variables which are highly skewed. Baseline characteristics of study population will be presented as means (standard deviation), median (inter-quartile ranges) or proportion according to the treatment groups. The difference in means among treatment groups will be compared using the analysis of variance (ANOVA). The difference in percentage treatment groups will be compared using the Chi-square test. If significant difference was found, post hoc test will be performed.

Missing data imputation: The analysis will be conducted under the intent-to-treat (ITT) principle. Under the ITT principle, all patients who are randomized will be included in the analysis. Therefore, missing data can be problematic. We will impute missing data using multiple imputation.[55]

Adjustment for multiplicity: A closed test procedure with the following sort order will be used for the comparisons. The second hypothesis will be tested only if the at least one first hypothesis has been rejected, thus maintaining the overall significance level at 5%.
Step 1:

1.1 The comparison of AM-PAC between Alter G and standard care (two-sided, alpha = 0.016)
1.2 The comparison of AM-PAC between PENS and standard care (two-sided, alpha = 0.016)
1.3 The comparison of AM-PAC between Alter G^+ and standard care (two-sided, alpha = 0.016)

Step 2: Perform various pairwise comparisons of AM-PAC if at least one comparison at step 1 will be significant. (Stepwise Holm's test)

Step 3: Perform various pairwise comparisons on secondary endpoints if at least one comparison at step 2 will be significant. (Stepwise Holm's test)

Special aim: The analysis will be conducted under the intent-to-treat (ITT) principle. All analysis will be stratified by study center. The primary endpoint will be the change in AM-PAC from baseline to end of the 12-week outpatient program. Analysis of Covariance (ANCOVA) will be used to test the difference in AM-PAC between the treatment groups adjusted for age, gender, baseline AM-PAC and other potential confounders. Furthermore, we will examine the difference in AM-PAC between the treatment groups over time. The mean (SD) of AM-PAC will be calculated at each time point. A plot of mean AM-PAC for each group versus time will be used to examine in visualizing whether the treatment groups and/or time have a significant effect on the mean AM-PAC. In addition, mixed models will be used to examine the longitudinal relationship between AM-PAC and treatment groups. Mixed model will incorporate random subject effect to account for the repeated observation will be used. To examine the possible interactions of treatment groups with age, gender, race and time, we will include the product of the factors in a discrete model to examine the possible moderators. For those endpoints that are binary, risk ratios will be calculated with use of logistic regression model and generalized mixed model.

Also, if applicable, we propose to use survival analysis, specifically the Kaplan-Meier method with Log-rank test, to test the difference in the curve of the proportion of patients being discharged, namely, achieving their treatment goals, during the 12-week treatment period across four groups.

Data Collection

Data for the proposed study will come from three main sources: (1) elements captured at time of initial evaluation and intervention, (2) repeated outcome measures and (3) follow-up questionnaire.

We will collect data using two main data capturing tools:
(1) REDCap (research electronic data capture: http://www.project-redcap.org) and
(2) AM-PAC

REDCap (http://www.project-redcap.org)

REDCap is a secure web application designed to assist data capture for research studies. There are over 312 institutions in the REDCap consortium. MedStar Health Research Institute has recently launched a MHRI REDCap application. The REDCap not only provides a web-based, user friendly platform to design and develop a data collection protocol for a research study, it also has a feature to allow study team to develop a customized survey. Most importantly, the MHRI REDCap application is secure and complies with federal and institutional ethical standards. REDCap is ideal for multi-site research collaborative in which it gives each participating site the freedom to access its respective data capture tools for data entry and survey implementation. The data can be exported in the formats used in major statistical packages, including SAS, SPSS, R, and STATA.

We will use REDCap to capture patient level data (e.g., demographics, impairment related data, comorbidities, intervention variables, and outcome measures; protocol to be developed by study team) other than functional level which will be measured and captured by a computer-based or a web-based AM-PAC (see below). We will also use REDCap to develop and implement the follow-up survey.

In addition, we will use the embedded function in REDCap to generate a Case Report Form for each patient enrolled in the proposed study.

AM-PAC

AM-PAC (Activity Measure for Post Acute Care) is a computerized adaptive testing instrument developed by Boston University Health and Disability Research Institute (BU HDRI) to measure functional level in 3 domains: basic mobility, daily activity, and applied cognition. In the proposed study, we propose to use a web-based AM-PAC for each participating site to access the instrument to measure study patient's functional level at multiple time points (see Measure and Instrument section). Pac-Metrix is the program that provides commercially available web-based AM-PAC instruments. See Appendix D for operations guide for Pac-Metrix. For the purpose of the study, we will only implement two domains in the AM-PAC, basic mobility and daily activity, as patients undergoing an elective TKA do not have any cognitive issue in general.

Patient Recruitment and Informed Consent Process

The number of eligible study participants will vary between 6 and 15 per month among the 13 study sites. See table below for estimated monthly patient volumes reported from each study site based on the number of TKA patients seen at each respective site in the previous year. We are confident that we are able to accomplish the goal of the proposed sample size (N=452, taking into account 20% attrition rate) in the proposed enrollment period:

Study Site	N of TKA/month	Study Site	N of TKA/month
OCOR	8-10	Bel Air	6-8
Oxon Hill	8-10	Perry Hall	8-10
Olney	10	Bethesda	8-10
Friendship Heights	10	Stadium Place	6-8
Sports Medicine at Lutherville	15	Salisbury	8-10
Harbor Hospital Sports Medicine	6-10	Ellicott City	8-10
McLean	Brand new facility; patient volume data not available at this time		

Patients who fulfill the inclusion and exclusion criteria will be eligible for the proposed study. A HIPAA authorization will be obtained so that the site coordinators could identify eligible patients upon outpatient physical therapy referral. The site coordinators will approach eligible patients and ask for written consent prior initial evaluation. The proposed study presents no more than a minimal risk to privacy as all electronic PHI is stored on a secure network (REDCap and NRH Rehabilitation Network) and is password protected. All newly enrolled patients' signed informed consents will be secured in locked file cabinets located in site coordinators' offices.

All researchers' human ethics training, including HIPAA privacy education and CITI training are current and valid. Each site coordinator will complete and obtain HIPAA privacy education and CITI training certificates prior to conducting any research.

Site coordinators will preferably be the clinical leads or directors (or their designee) at each participating site mainly because they have dedicated administrative time to (1) consent and enroll patients and (2) assist in local research operations such as data entry. Their responsibilities include, but not limited to:

1. Complete the required HIPAA privacy and CITI training module and obtain a PEER number prior IRB application.
2. Work with NRH-CPIR in obtaining IRB approval.
3. Attend a half-day or possibly a full-day training, followed by regular webinars for training on REDCap and AM-PAC, and by regular conference calls.
4. Identify eligible study patients per study selection and exclusion criteria and obtain their informed consent.
5. Assign study patients to one of the four treatment groups using randomization approach.
6. Maintain an enrollment log at local site.
7. Assist data collection using AM-PAC and REDCap for data entry.
8. Participate in study meetings/conference calls and data analyses.

Storage and Pooling of Data

All data collected through the REDCap and AM-PAC will be protected using a multi-layered security system, including an individualized password for each site coordinator to access both applications for data entry purpose. The REDCap administrator, Stephen Fernandez, and the Research Engineer of the proposed study, MJ Tang Hu will work together to assure data security. Both Mr. Fernandez and Ms. Hu will have the administrator authority to grant a user log-in and define the level of access for each site coordinator and the research team.

Ms. Hu will be responsible for periodic data compilation to merge data from the REDCap and AM-PAC for a complete study database for purpose of interim monitoring and final analysis. She will also be charged, working together with the research staff and site coordinators, to develop the follow-up survey in REDCap.

Risk/Benefits Assessment

Use of any medical devices involves risks. We will assure that physical therapists who are involved in the proposed study will receive adequate and necessary training to operate these devices, specifically, AlterG and PENS appropriately by following their respective instructional manuals. In addition, patient's safety is the top one priority in all MedStar National Rehabilitation Network as part of the standard clinical practice.

Whether or not patients are enrolled in the study, fatigue from the therapy session may occur probably in the first few visits mainly when patients may have limited exercise tolerance after their knee replacement procedure. If fatigue occurs, therapists will allow patients to rest and ask patients to resume therapy activity when they are able. When patients build up their strength and endurance after a couple of therapy visits, fatigue may no longer occur. Patients may

experience fatigue from a longer visit when they are asked to spend approximately 20 more minutes to do some study related, performance-based measures immediately following these therapy sessions (4 out of 24 sessions). Physical therapists will make sure patients' safety and let them rest. Similarly, chance of falling from increased weight bearing on patient's surgical side (leg) during therapy session is possible but less likely to occur. Preventions of falling are routinely in place in standard physical therapy practice whether or not patients are enrolled in the study. Like any other existing neuromuscular stimulation devices, the PENS uses electrodes to place on patient's skin. Skin irritation may occur. If skin irritation occurs, therapists will remove the electrode and turn off the PENS immediately and will evaluate if patients should be excluded from the study.

There is a very remote risk that Personal Health Information (PHI) could be disclosed. This will be monitored and prevented by following HIPAA guidelines and keeping all data secure.

Information about comparative effectiveness of outpatient physical therapy among patients with TKA will be collected during the study, resulting in more effective rehabilitation.

Compensation

There will be no compensation provided for study participants. Participation in the study is entirely voluntary. The study imposes no additional burden on the patient except for extra time for repeated outcome measures.

Modification of Protocol

Any modification to the protocol, consent form, and/or questionnaires, including a change to the PI, will be submitted to the MedStar IRB for review and approval. Major modifications to the research protocol and any modifications that could increase risk to volunteers will be submitted to the MedStar IRB for approval prior to implementation.

Protocol Deviations

Any deviation to the protocol that may have an effect on the safety or rights of the subject or the integrity of the study will be reported to the MedStar IRB.

Reporting of Serious Adverse Events and Unanticipated Problems

Unanticipated problems involving risk to volunteers or others, serious adverse events related to participation in the study and all volunteer deaths related to participation in the study should be promptly reported to the MedStar IRB. A complete written report will follow the initial notification.

Data Collection Flow Chart

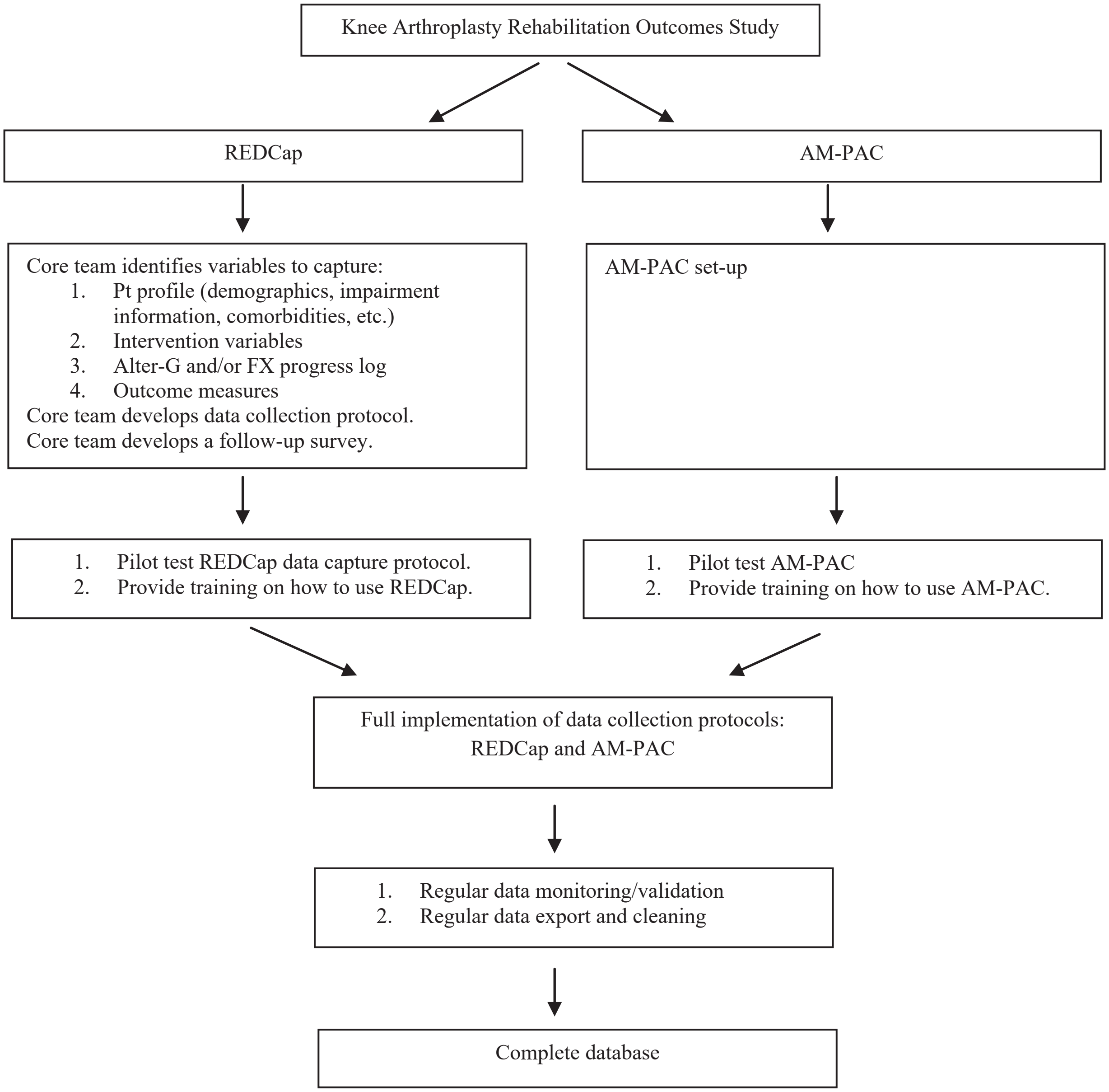

Study Flow Diagram

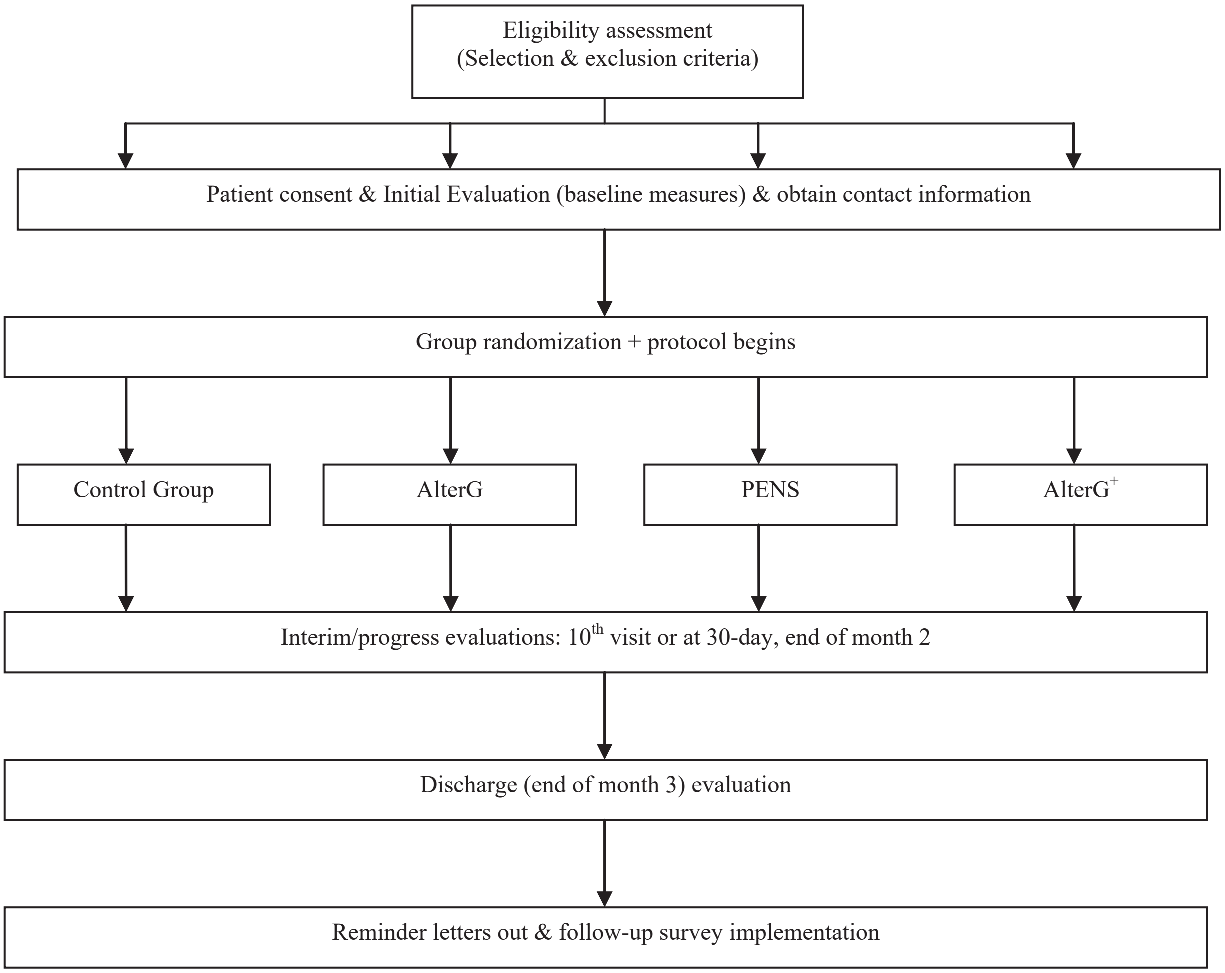

Project Timeline

	2012			2013		2013-2014
**Project Task**	**Oct.**	**Nov..**	**Dec.**	**Jan.-Mar.**	**Apr.-Sept.**	**Oct.-Sept.**
Submit and obtain scientific review board (SRB) approval	x					
Submit and obtain IRB approval	x	x	x			
Develop data collection protocol (REDCap)			x	x		
Pilot test study protocol				x		
Finalize study protocol			x			
Prepare data collection				x		
Develop and pilot test follow-up survey				x	x	
Submit IRB amendment for follow-up survey				x	x	
Prepare for and conduct training sessions			x	x		
Begin enrollment and data collection				x	x	x
Conduct interim reviews and prepare for interim reports				x	x	x
Conduct data analyses				x	x	x
Prepare for disseminations and publications					x	x

REFERENCES

[1] Tian W, DeJong G, Brown M, et al. Looking upstream: factors shaping the demand for postacute joint replacement rehabilitation. *Arch Phys Med Rehabil* 2009;90:1260-8.

[2] Harada N, Chiu V, Damron-Rodriguez J, et al. Screening for balance and mobility impairment in elderly individuals living in residential care facilities. *Phys Ther* 1995;75:462-469.

[3] Perry J, Garrett M, Gronley JK, Mulroy SJ. Classification of walking handicap in the stroke population. *Stroke* 1995;26:982-989.

[4] Leslie LR. Training for functional independence. In: Kottke FJ, Lehmann JF, eds. *Krusen's Handbook of Physical Medicine and Rehabilitation*. 4th ed. Philadelphia, Pa: WB Saunders Co; 1990:564-570.

[5] Hoffer MM, Feiwell E, Perry R, Perry J, Bonnett C. Functional ambulation in patients with myelomeningocele. *J Bone Joint Surg Am* 1973;5:137-148.

[6] Guralnik JM, Simonsick EM, Ferrucci L, et al. A short physical performance battery assessing lower extremity function: association with self-reported disability and prediction of mortality and nursing home admission. *J Gerontol* 1994;49:M85-94.

[7] Guralnik JM, Ferrucci L, Pieper CF, et al. Lower extremity function and subsequent disability: consistency across studies, predictive models, and value of gait speed along compared with the short physical performance battery. *J Gerontol A Biol Sci Med Sci* 2000;55A:M221-231.

[8] Bean JF, Kiely DK, LaRose S, et al. Is stair climb power a clinically relevant measure of leg power impairments in at-risk older adults? *Arch Phys Med Rehabil* 2007;88:604-9.

[9] Hardy SE, Perera S, Roumani YF, et al. Improvement in usual gait speed predicts better survival in older adults. *J Am Geriatr Soc* 2007;55:1727-1734.

[10] Brown CJ, Bradberry C, Howze SG, et al. Defining community ambulation from the perspective of the older adult. *J Geriatr Phys Ther* 2010;33:56-63.

[11] Dennett AM, Taylor NF, Mulrain K. Community ambulation after hip fracture: completing tasks to enable access to common community venues. *Disabil Rehabil* 2012;34:707-714.

[12] DeJong G, Horn SD, Smout RJ, et al. Joint replacement rehabilitation outcomes on discharge from skilled nursing facilities and inpatient rehabilitation facilities. *Arch Phys Med Rehabil* 2009;90:1284-96.

[13] Jette D, Warren R, Wirtalla C. The relation between therapy intensity and outcomes of rehabilitation in skilled nursing facilities. *Arch Phys Med Rehabil* 2005;86:373-9.

[14] Munin M, Rudy T, Glynn N, et al. Early inpatient rehabilitation after elective hip and knee arthroplasty. *JAMA* 1998;279:847-52.

[15] Dobkin B, Barbeau H, Deforge D, et al. The evolution of walking-related outcomes over the first 12 weeks of rehabilitation for incomplete traumatic spinal cord injury: the multicenter randomized spinal cord injury locomotor trial. *Neurorehabil Neural Repair* 2007;21"25-35.

[16] Dobkin B, Apple D, Barbeau H, et al. Methods for a randomized trial of weight-supported treadmill training versus conventional training for walking during inpatient rehabilitation after incomplete traumatic spinal cord injury. *Neurorehabil Neural Repair* 2003;17:153-167.

[17] Sullivan KJ, Knowlton BJ, Dobkin BH. Step training with body weight support: effect of treadmill speed and practice paradigms on poststroke locomotor recovery. *Arch Phys Med Rehabil* 2002;83:683-691.

[18] Toole T, Maitland CG, Warren E, et al. The effects of loading and unloading treadmill walking on balance, gait, fall risk, and daily function in Parkinsonism. *NeuroRehabilitation* 2005;20:307-322.

[19] Mossberg KA, Orlander KE, Norcross JL. Cardiorespiratory capacity after weight-supported treadmill training in patients with traumatic brain injury. *Phys Ther* 2008;88:77-87.

[20] Kurz M, Corr B, Stuberg W, et al. Evaluation of lower body positive pressure supported treadmill training for hildren with cerebral palsy. *Pediatr Phys Ther* 2011;23:232-9.

[21] Eastlack RK, Hargens AR, Groppo ER, et al. Lower body positive-pressure exercise after knee surgery. *Clin Orthop Relat Res* 2005;431:213-9.

[22] Saxena A, Granot A. Use of an anti-gravity treadmill in the rehabilitation of the operated Achilles tendon: a pilot study. *J Foot and Ankle Surgery* 2011;50:558-561.

[23] Gotlin RS, Hershkowitz S, Juris PM, et al. Electrical stimulation effect on extensor lag and length of hospital stay after total knee arthroplasty. *Arch Phys Med Rehabil* 1994;75:957-959.

[24] Snyder-Mackler L, Delitto A, Bailey SL, Stralka SW. Use of electrical stimulation to enhance recovery of quadriceps femoris muscle force production in patients following anterior cruciate ligament reconstruction. Phys Ther 1994;74:901-907.

[25] Avramidis K, Strike PW, Taylor PN, Swain ID. Effectiveness of electric stimulation of the vastus medialis muscle in the rehabilitation of patients after total knee arthroplasty. *Arch Phys Med Rehabil* 2003;84:1850-1853.

[26] Stevens JE, Mizner RL, Snyder-Mackler L. Neuromuscular electrical stimulation for quadriceps muscle strengthening after bilateral total knee arthroplasty: a case series. *J Orthop Sports Phys Ther* 2004;34:21-29.

[27] Mintken PE, Carpenter KJ, Eckhoff D, et al. Early neuromuscular electrical stimulation to optimize quadriceps muscle function following total knee arthroplasty: a care report. *J Orthop Sports Phys Ther* 2007;37:364-371.

[28] McAlister FA, Straus SE, Sackett DL, Altman DG. Analysis and reporting of factorial trials: a systematic review. *JAMA* 2003 May;289(19):2545-2553.

[29] Jette A, Haley SM, Tao W, et al. Prospective evaluation of the AM-PAC in outpatient rehabilitation settings. *Phys Ther* 2007;87:385-398.

[30] Lantham NK, Mehta V, Nguyen AM, et al. Performance-based or self-report measures of physical function: which should be used in clinical trials of hip fracture patients? *Arch Phys Med Rehabil* 2008;89:2146-55.

[31] Balk B. A simple field test for the assessment of physical fitness. Rep Civ Aeromed Res Inst US. 1963(53):1-8.

[32] Troosters T, Gosselink R, Decramer M. Six minute walking distance in healthy elderly subjects. *Eur Respir J.* 1999 Aug;14(2):270-274.

[33] Harada ND, Chiu V, Stewart AL. Mobility-related function in older adults: assessment with a 6-minute walk test. *Arch Phys Med Rehabil*. 1999 Jul;80(7):837-41.

[34] Duncan PW, et al. Protocol for the locomotor experience applied post-stroke (LEAPS) trial: a randomized controlled trial. *BMC Neurology*; 7(1):39.

[35] Enright PL, et al. (1998). Reference equations for the six-minute walk in healthy adults. *Am J Respir Crit Care Med*; 158(5pt1):1384-1387.

[36] Guyatt GH, et al. The 6-minute walk: a new measure of exercise capacity in patients with chronic heart failure. *Can Med Assoc J.* 1985;132(8):919-923.

[37] Holden MK, et al. Gait assessment for neurologically impaired patients. Standards for outcome assessment. *Phys Ther.* 1986;66(10):1530-1539.

[38] Pohl P, et al. Influence of stroke-related impairments in performance in 6-minute walk test. *J Rehabil Res Dev*. 2002;39:439-444.

[39] Crosbie J, Naylor J, Harmer A, Russell T. Predictor of functional ambulation and patient perception following total knee replacement and short-term rehabilitation. *Disability and Rehabilitation*, 2010;32(13):1088-1098.

[40] French HP, Fitzpatrick M, FitzGerald O. Responsiveness of physical function outcomes following physiotherapy intervention for osteoarthritis of the knee: an outcome comparison study. *Physiotherapy*, 2011;97(4):302-8.

[41] Parent E, Moffet H. Comparative responsiveness of locomotor tests and questionnaires used to follow early recovery after total knee arthroplasty. *Arch Phys Med Rehabil* 2002;83:70-80.

[42] Mizner RL, Patterson SC, Clements KE, Zeni JA, et al. Measuring functional improvement after total knee arthroplasty requires both performance-based and patient-report assessments. *The Journal of Arthroplasty*, 2011 Aug;26(5):728-37.

[43] Fritz S, Lusardi M. White paper: "walking speed: the sixth vital sign." *Journal of Geriatric Physical Therapy* 2009;32:2-5.

[44] Steffen TM, Hacker TA, Mollinger L. Age- and gender-related test performance in community-dwelling elderly people: Six-Minute Walk Test, Berg Balance Scale, Timed Up & Go Test, and gait speeds. Phys Ther 2002;82:128-137.

[45] Studenski S, Perera S, Wallace D, et al. Physical performance measures in the clinical setting. *J Am Geriatr Soc* 2003;51:314-322.

[46] Bohannon RW. Comfortable and maximum walking speed of adults aged 20-79 years: reference values and determinants. *Age Ageing* 1997;26:15-19.

[47] Lusardi M, Pellecchia G, Schulman M. Functional performance in community living older adults. *J Geriatr Phys Ther* 2003;26:14-22.

[48] Schmid A, Duncan PW, Studenski S, et al. Improvements in speed-based gait classifications are meaningful. *Stroke* 2007;38:2096-2100.

[49] Bowden MG, Balasubramanian CK, Behrman AL, Kautz SA. Validation of a speed-based classification system using quantitative measures of walking performance poststroke. *Neurorehabil Neural Repair* 2008;22:672-675.

[50] Roos EM, Lohmander LS. The knee injury and osteoarthritis outcome score (KOOS): from joint injury to osteoarthritis. *Health and Quality of Life Outcomes*, 2003;1:64-82.

[51] Bellamy N, Buchanan WW, Goldsmith CH, Campbell J and Stitt LW. Validation study of WOMAC: a health status instrument for measuring clinically important patient relevant outcomes to antirheumatic drug therapy in patients with osteoarthritis of the hip or knee. *J Rheumatol* 1988, 15:1833-1840.

[52] www.koos.nu Accessed December 20, 2011.

[53] http://www.jointcommission.org/assets/1/18/Pain_Management.pdf

[54] DeJong G, Hsieh CH, Gassaway J, et al. Characterizing rehabilitation services for patients with knee and hip replacement in skilled nursing facilities and inpatient rehabilitation facilities. *Arch Phys Med Rehabil* 2009;90:1269-83.

[55] Rubin DB. 1987. Multiple imputation for nonresponse in surveys. John Wiley & Sons, New York.
