# Supplementary material for: Effect of Outpatient Rehabilitation on Functional Mobility After Single Total Knee Arthroplasty: A Randomized Clinical Trial
Source: JAMA Netw Open. 2020 Sep 17;3(9):e2016571. doi: 10.1001/jamanetworkopen.2020.16571 (PMC7499127; doi:10.1001/jamanetworkopen.2020.16571)
Supplement: Supplement 2. — eTable 1. AM-PAC and 6-Minute Walk Test Linear Mixed Model Coefficients and Interactions eFigure 1. AM-PAC Scores Over Time eFigure 2. 6-Minute Walk Test Performances Over Time eTable 2. AM-PAC and 6-Minute Walk Test Scores Between Early and Later Starters [file jamanetwopen-e2016571-s002.pdf]

Supplementary Online Content

Hsieh CJ, DeJong G, Vita M, Zeymo A, Desale S. Effect of outpatient rehabilitation on functional mobility after single total knee arthroplasty: a randomized clinical trial. *JAMA Netw Open*. 2020;3(9):e2016571. doi:10.1001/jamanetworkopen.2020.16571

eTable 1. AM-PAC and 6-Minute Walk Test Linear Mixed Model Coefficients and Interactions

eFigure 1. AM-PAC Scores Over Time

eFigure 2. 6-Minute Walk Test Performances Over Time

eTable 2. AM-PAC and 6-Minute Walk Test Scores Between Early and Later Starters

This supplementary material has been provided by the authors to give readers additional information about their work.

© 2020 Hsieh CJ et al. *JAMA Network Open.*

eTable 1. AM-PAC and 6-Minute Walk Test Linear Mixed Model Coefficients and Interactions

	Interventions			
	**Standard of Care: Recumbent Bike (Control) (N=92)**	**Body weight-adjustable treadmill (Intervention 1) (N=91)**	**Recumbent Bike + PENS (Intervention 2) (N=90)**	**Both (Intervention 3) (N=90)**
**AMPAC**				
Baseline	Reference	-1.1 (0.19)	-0.4 (0.64)	-0.63 (0.46)
Time Effect*				
1st repeated evaluation	6.06 (<0.001)	1.2 (0.22)	-0.13 (0.90)	0.63 (0.52)
2nd repeated Evaluation	8.46 (<0.001)	1.4 (0.18)	0.94 (0.39)	1.0 (0.35)
3rd repeated Evaluation	10.34 (<0.001)	-0.26 (0.86)	2.27 (0.14)	0.86 (0.58)
**6MWT**				
Baseline	Reference	-5.0 (0.75)	-14.6 (0.35)	-17.4 (0.27)
Time Effect*				
1st repeated evaluation	118.9 (<0.001)	-8.71 (0.55)	-13.8 (0.34)	-18.0 (0.21)
2nd repeated Evaluation	149.1 (<0.001)	-6.57 (0.67)	8.15 (0.62)	35.9 (0.02)
3rd repeated Evaluation	176.7 (<0.001)	-24.2 (0.27)	26.9 (0.24)	20.4 (0.37)

Models control for age, gender, BMI, course of outpatient treatment, acute length of stay, and number of outpatient visits.

*: Time effects in the control column represent the time main effects while the time effects in the other columns represent the interaction between time and intervention.

Numbers in the parentheses are the p values.

The linear mixed modeling, as shown in this eTable 1 confirmed the results comparing outcomes, both AMPAC and 6MWT, as shown in Table 3. Coefficients for time main effect are statistically significant and monotonic, indicating improved AMPAC and 6MWT scores at later evaluations. The interaction terms (time x intervention) captured the differences in the improvement (AMPAC and 6MWT respectively) between each intervention relative to the control group.

© 2020 Hsieh CJ et al. *JAMA Network Open.*

eFigure 1. AM-PAC Scores Over Time

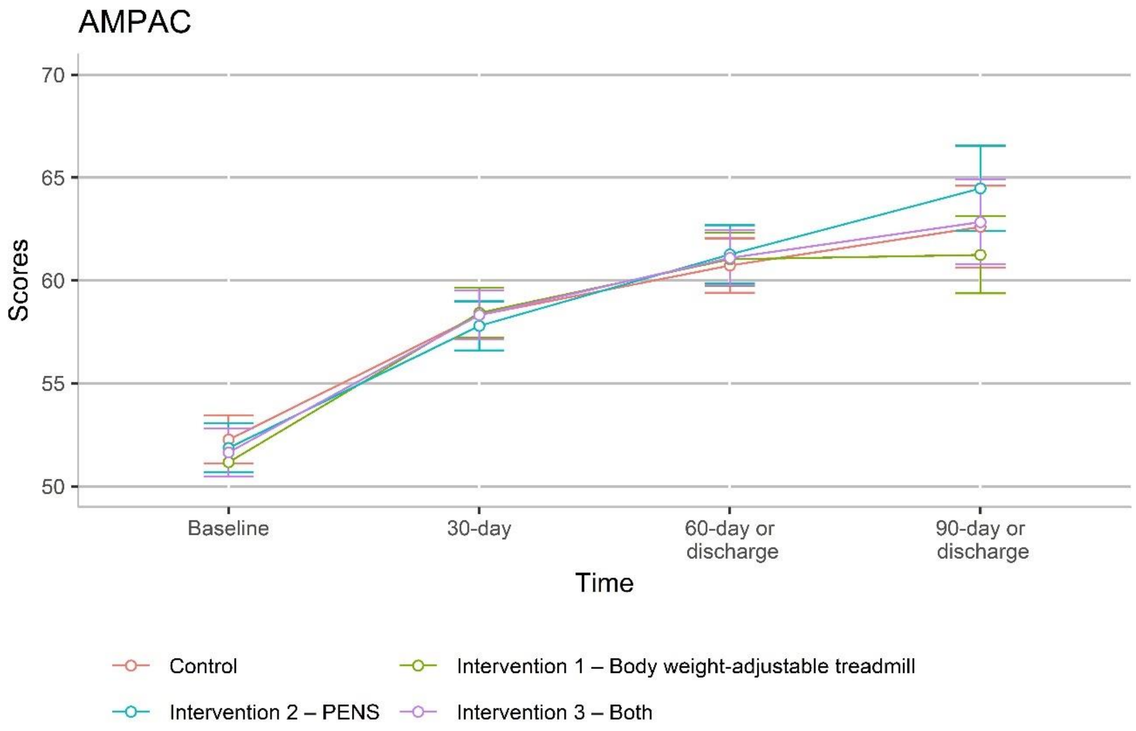

eFigure 1 presents the estimated AMPAC scores over time from baseline to discharge, adjusting for age, gender, BMI, course of outpatient treatment, acute length of stay, and number of outpatient visits. The recovery trajectory in terms of functional outcome as measured by AMPAC is comparable across groups.

© 2020 Hsieh CJ et al. *JAMA Network Open.*

eFigure 2. 6-Minute Walk Test Performances Over Time

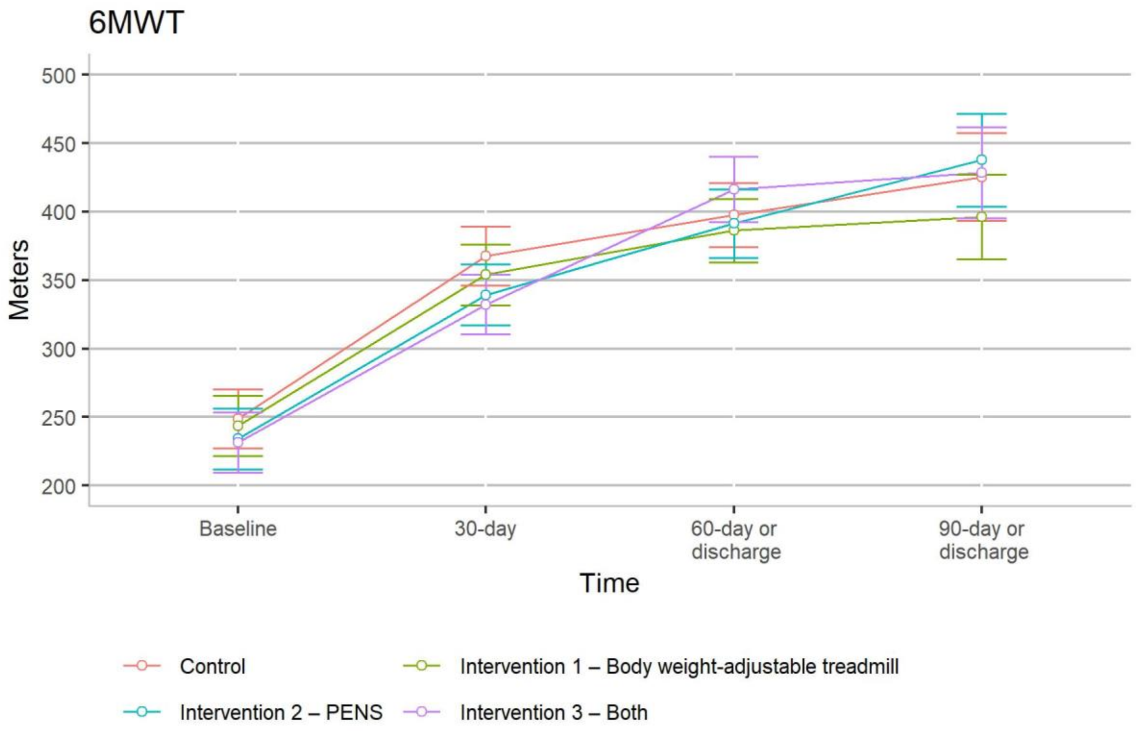

eFigure 2 presents the estimated 6MWT over time from baseline to discharge, adjusting for age, gender, BMI, course of outpatient treatment, acute length of stay, and number of outpatient visits. The improved outcome in terms of functional capacity as measured by 6MWT is comparable across groups.

© 2020 Hsieh CJ et al. *JAMA Network Open.*

eTable 2. AM-PAC and 6-Minute Walk Test Scores Between Early and Later Starters

eTable 2a: AMPAC Scores between Early and Later Starters

	**Standard of Care: Recumbent Bike**<br><br>**Control (N=92)**	**Body weight-adjustable treadmill**<br><br>**(Intervention 1) (N=91)**	**Recumbent Bike + PENS**<br><br>**(Intervention 2) (N=90)**	**Both**<br><br>**(Intervention 3) (N=90)**	***P* value**
**At outpatient initial evaluation**					
AMPAC for early starters (mean, SD)*(95% CIs)*	N=25<br>48.8 (7.0)<br>*(46.1, 51.5)*	N=27<br>49.0 (6.2)<br>*(46.7, 51.3)*	N=22<br>51.4 (8.9)<br>*(47.7, 55.1)*	N=26<br>49.5 (6.9)<br>*(46.8, 52.2)*	0.60
AMPAC for later starters (mean, SD)*(95% CIs)*	N=67<br>53.7 (5.3)<br>*(52.4, 55.0)*	N=64<br>52.2 (6.9)<br>*(50.5, 53.9)*	N=68<br>52.0 (4.5)<br>*(50.9, 53.1)*	N=64<br>52.6 (5.5)<br>*(51.3, 53.9)*	0.30
**At discharge from outpatient**					
AMPAC for early starters (mean, SD)*(95% CIs)*	N=25<br>65.3 (5.1)<br>*(63.3, 67.3)*	N=27<br>60.5 (5.0)<br>*(58.6, 62.4)*	N=22<br>61.1 (7.9)<br>*(57.8, 64.4)*	N=26<br>62.2 (5.5)<br>*(60.1, 64.3)*	0.02
AMPAC for later starters (mean, SD)*(95% CIs)*	N=67<br>59.8 (4.6)<br>*(58.7, 60.9)*	N=64<br>61.6 (5.6)<br>*(60.2, 63.0)*	N=68<br>61.1 (5.5)<br>*(59.8, 62.4)*	N64<br>60.7 (6.7)<br>*(59.1, 62.3)*	0.34
**Improvement from initial evaluation to discharge**					
AMPAC for early starters (mean, SD)*(95% CIs)*	N=25<br>16.5 (9.4)<br>*(12.8, 20.2)*	N=27<br>11.5 (6.9)<br>*(8.9, 14.1)*	N=22<br>9.7 (8.1)<br>*(6.3, 13.1)*	N=26<br>12.7 (8.5)<br>*(9.4, 16)*	0.04
AMPAC for later starters (mean, SD)*(95% CIs)*	N=67<br>6.2 (6.1)<br>*(4.7, 7.7)*	N=64<br>9.4 (7.5)<br>*(7.6, 11.2)*	N=68<br>9.1 (5.7)<br>*(7.7, 10.5)*	N=64<br>8.1 (6.2)<br>*(6.6, 9.6)*	0.02

Early vs. later starter groups:

We found a clear bimodal distribution among early vs. later starters in the study sample (N=363): Early starters were individuals who started their outpatient therapy ≤ 9 days and later starters were those who started outpatient therapy ≥ 10 days post-TKA. This distribution was driven, in part, by post-TKA referral patterns: some came directly to outpatient care (early starters) and others received a period of home health care prior to outpatient therapy (later starters).

As expected, early starters, because they started from lower baseline, made larger gains between baseline and discharge compared to later starters across all 4 intervention groups. Interestingly, early starters had equal or better functional outcomes (as measured by AMPAC) than later starters within each intervention upon discharge (unadjusted for potential differences between early and later starters). Note that early starters in the control and Intervention 3 groups improved the most upon discharge in comparison with the other two groups. Given the relatively small N in the early starter groups, we caution readers not to make any statistical or clinical inferences. In addition, the cut-off threshold of 9 days used here may not be sufficiently sensitive in detecting the optimal moment at which to incorporate any of the devices used in the study in order to facilitate a faster recovery or yield a better outcome post-TKA.

© 2020 Hsieh CJ et al. *JAMA Network Open.*

eTable 2b: 6MWT between Early and Later Starters

	Standard of Care: Recumbent Bike<br><br>Control (N=92)	Body weight-adjustable treadmill<br><br>(Intervention 1) (N=91)	Recumbent Bike + PENS<br><br>(Intervention 2) (N=90)	Both<br><br>(Intervention 3) (N=90)	*P* value
**At outpatient initial evaluation**					
6MWT for early starters (meters, mean, SD)*(95% CIs)*	N=25<br>207.6 (88.6)<br>*(172.9, 242.3)*	N=27<br>203.8 (94.9)<br>*(168.0, 239.6)*	N=22<br>221.6 (89.5)<br>*(184.2, 259.0)*	N=26<br>173.4 (71.1)<br>*(146.1, 200.7)*	0.26
6MWT for later starters (meters, mean, SD)*(95% CIs)*	N=66<br>264.1 (87.7)<br>*(242.9, 285.3)*	N=64<br>257.5 (100.2)<br>*(233.0, 282.0)*	N=68<br>264.2 (335.4)<br>*(184.5, 343.9)*	N=63<br>247.9 (97.4)<br>*(223.8, 272.0)*	0.96
**At discharge from outpatient**					
6MWT for early starters (meters, mean, SD)*(95% CIs)*	N=25<br>430.0 (94.5)<br>*(393.0, 467.0)*	N=27<br>384.2 (135.1)<br>*(333.2, 435.2)*	N=22<br>438.5 (131.8)<br>*(383.4, 493.6)*	N=26<br>397.2 (97.0)<br>*(359.9, 434.5)*	0.30
6MWT for later starters (meters, mean, SD)*(95% CIs)*	N=67<br>394.9 (111.5)<br>*(368.2, 421.6)*	N=64<br>386.3 (125.7)<br>*(355.5, 417.1)*	N=68<br>425.5 (516.1)<br>*(302.8, 548.2)*	N=62<br>394.4 (109.0)<br>*(367.3, 421.5)*	0.86
**Improvement from initial evaluation to discharge**					
6MWT for early starters (meters, mean, SD)*(95% CIs)*	N=25<br>222.4 (73.6)<br>*(193.5, 251.3)*	N=27<br>180.5 (81.7)<br>*(149.7, 211.3)*	N=22<br>216.9 (150.9)<br>*(153.8, 280.0)*	N=26<br>223.8 (122.1)<br>*(176.9, 270.7)*	0.43
6MWT for later starters (meters, mean, SD)*(95% CIs)*	N=66<br>130.3 (91.5)<br>*(108.2, 152.4)*	N=64<br>128.8 (121.0)<br>*(99.2, 158.4)*	N=68<br>161.2 (209.7)<br>*(111.4, 211.0)*	N=62<br>145.2 (107.0)<br>*(118.6, 171.8)*	0.52

Early vs. later starter groups are defined the same as those in eTable 2a.

As expected, early starters, because they started from lower baseline, made larger 6MWT gains between baseline and discharge compared to later starters within each intervention; early starters within each intervention group had equal or better functional outcomes (as measured by 6MWT) than later starters within each intervention upon discharge.

Given the relatively small N in the early starter groups, we caution readers not to make any statistical or clinical inferences. In addition, the cut-off threshold of 9 days used here may not be sufficiently sensitive in detecting the optimal moment at which to incorporate the devices examined in the study in order to facilitate a faster recovery or yield a better outcome post-TKA.

Moreover, given the overlapping confidence intervals across the 4 interventions, no intervention stood out as producing superior 6MWT outcomes, neither among early nor later starters.

© 2020 Hsieh CJ et al. *JAMA Network Open.*
