# Supplementary material for: Effect of Outpatient Rehabilitation on Functional Mobility After Single Total Knee Arthroplasty: A Randomized Clinical Trial
Source: JAMA Netw Open. 2020 Sep 17;3(9):e2016571. doi: 10.1001/jamanetworkopen.2020.16571 (PMC7499127; doi:10.1001/jamanetworkopen.2020.16571)
Supplement: Supplement 3. — Data Sharing Statement [file jamanetwopen-e2016571-s003.pdf]

Data Sharing Statement

Hsieh. Effect of Outpatient Rehabilitation on Functional Mobility After Single Total Knee Arthroplasty. *JAMA Netw Open*. Published September 17, 2020. 10.1001/jamanetworkopen.2020.16571

Data
Data available: No

Additional Information
Explanation for why data not available: We continue to conduct secondary analyses using the same dataset, therefore, decided not to share the data at this time.
